# c-Src controls stability of sprouting blood vessels in the developing retina independently of cell-cell adhesion through focal adhesion assembly

**DOI:** 10.1242/dev.185405

**Published:** 2020-04-06

**Authors:** Lilian Schimmel, Daisuke Fukuhara, Mark Richards, Yi Jin, Patricia Essebier, Emmanuelle Frampton, Marie Hedlund, Elisabetta Dejana, Lena Claesson-Welsh, Emma Gordon

**Affiliations:** 1 Institute for Molecular Bioscience, The University of Queensland, Brisbane, Queensland 4072, Australia; 2 Uppsala University, Beijer and Science for Life Laboratories, Department of Immunology, Genetics and Pathology, Rudbeck Laboratory, Uppsala 75185, Sweden

**Keywords:** Angiogenesis, c-Src, Adherens junctions, Focal adhesions, Kinase signaling

## Abstract

Endothelial cell adhesion is implicated in blood vessel sprout formation, yet how adhesion controls angiogenesis, and whether it occurs via rapid remodeling of adherens junctions or focal adhesion assembly, or both, remains poorly understood. Furthermore, how endothelial cell adhesion is controlled in particular tissues and under different conditions remains unexplored. Here, we have identified an unexpected role for spatiotemporal c-Src activity in sprouting angiogenesis in the retina, which is in contrast to the dominant focus on the role of c-Src in the maintenance of vascular integrity. Thus, mice specifically deficient in endothelial c-Src displayed significantly reduced blood vessel sprouting and loss in actin-rich filopodial protrusions at the vascular front of the developing retina. In contrast to what has been observed during vascular leakage, endothelial cell-cell adhesion was unaffected by loss of c-Src. Instead, decreased angiogenic sprouting was due to loss of focal adhesion assembly and cell-matrix adhesion, resulting in loss of sprout stability. These results demonstrate that c-Src signaling at specified endothelial cell membrane compartments (adherens junctions or focal adhesions) control vascular processes in a tissue- and context-dependent manner.

## Abstract (precis)

**Summary:** c-Src is required for developmental angiogenic sprouting in the retina by controlling cell-matrix adhesion, revealing a context-dependent role for kinase signaling in endothelial cells.

## INTRODUCTION

Blood vessels form complex branched networks comprising arteries, capillaries and veins that supply oxygen and nutrients to all body tissues. Vascular outgrowth occurs through a process called sprouting angiogenesis, which is essential for laying down a functional vessel network during embryonic development. Sprouting is also initiated later in life in response to environmental changes such as tissue injury and growth, as well as in pathologies such as cancer and eye disease (Potente et al., 2011). A common denominator for these processes is hypoxia, which drives expression of vascular endothelial growth factors (VEGFs), acting to initiate sprouting through binding and activation of VEGF receptors (VEGFRs). Signaling downstream of these ligand/receptor complexes is essential for vascular morphogenesis, as they control processes such as endothelial cell (EC) identity, migration, proliferation and vessel permeability (Simons et al., 2016). VEGFA/VEGFR downstream signaling converges with that of integrins, which control multiple endothelial cell processes by binding to a range of ligands, including extracellular matrix (ECM) proteins (Hynes et al., 2002). A greater understanding of the exquisite specificity of downstream signaling required for angiogenesis is now becoming appreciated, in order to precisely target specific vascular processes in disease.

During sprout formation and elongation, ECs respond to instructive cues in a collective manner while displaying heterogeneous gene expression, morphology and behavior. During blood vessel formation, the importance of differential adhesion between ECs in the growing sprout has been described from studies in mouse and zebrafish (Bentley et al., 2014; Lenard et al., 2013). Differential cell-cell adhesion is modulated by the junctional localization of the main endothelial adhesion molecule vascular endothelial cadherin (VE-cadherin), the internalization of which is dependent on phosphorylation at distinct tyrosine sites within its intracellular tail (Gordon et al., 2016; Orsenigo et al., 2012; Wessel et al., 2014). In quiescent cell monolayers or *ex vivo* models, VE-cadherin is neither phosphorylated nor internalized, leading to excessively strong cell-cell adhesions, thereby inhibiting EC migration and sprout formation (Bentley et al., 2009). Conversely, in high VEGFA signaling scenarios, such as in cancer, VE-cadherin phosphorylation and internalization is exaggerated, resulting in impaired adhesiveness and formation of non-functional vessels (Bentley et al., 2009). *In vivo*, VE-cadherin phosphorylation is controlled by VEGF and hemodynamic forces through a mechanosensory complex composed of VEGFR2/VEGFR3, PECAM and c-Src (Src - UNIProt) (Conway et al., 2013, 2017; Coon et al., 2015; Orsenigo et al., 2012; Tzima et al., 2005). There is still some uncertainty about the conditions under which various signals mediate VE-cadherin dynamics, and how this controls barrier properties remains largely unknown.

VEGFR2 contains a number of tyrosine residues within its intracellular domain that are differentially phosphorylated upon VEGFA stimulation (Koch and Claesson-Welsh, 2012). Each of these individual phosphorylation sites have specific interaction partners and the *in vivo* roles of each of these are now being unraveled. Cell-cell adhesion in the blood vasculature has been identified as being downstream of the VEGFR2 Tyr949 site (Tyr951 in the human), through the *VEGFR2-949/TSAd/c-Src/VE-cadherin* cascade. Phosphorylation of the Tyr949 residue in mouse VEGFR2 by VEGFA mediates binding of the T-cell specific adaptor (TSAd) protein, which is essential for activation of c-Src at cell-cell junctions (Matsumoto et al., 2005). TSAd is devoid of intrinsic kinase activity, but acts as a scaffold to recruit c-Src to junctions. Active c-Src at EC junctions can phosphorylate VE-cadherin and mediate its internalization, thereby lowering the pool of VE-cadherin available to engage in adhesion, promoting increased leakage from the blood vessel, which is known as vascular permeability (Sun et al., 2012). In addition to controlling vascular permeability, VEGFR2-949/TSAd/c-Src/VE-cadherin signaling is crucial for sprouting angiogenesis in certain tissues. The presence of TSAd/c-Src at cell-cell junctions, accompanied by VE-cadherin phosphorylation and internalization, is required for sprouts to elongate in the trachea (Gordon et al., 2016). Thus, it is known that c-Src exists at junctions (Orsenigo et al., 2012), yet a second subcellular pool has also been identified at focal adhesions (FAs) (Westhoff et al., 2004). It is conceivable that the different subcellular pools of c-Src are controlled by different pathways and, depending on the instructive cues and surrounding environment, they ultimately lead to phosphorylation of distinct sets of c-Src substrates regulating cell-cell (junctions) or cell-matrix (focal adhesions) dynamics.

Although we have previously identified a role for TSAd/c-Src in sprout elongation (Gordon et al., 2016), a role for c-Src in angiogenesis has remained unsettled. In the 1990s it was reported that mice with a global deletion of either *Src* or the related c-Src family kinases (SFKs) *Yes* and *Fyn* have normal sprouting angiogenesis, but display abnormal vessel barrier integrity (Eliceiri et al., 1999). Indeed, in mature vessels of adult mice, SFKs can induce VE-cadherin phosphorylation at Tyr658 and Tyr685 in veins but not arteries, which is necessary, but not sufficient, to induce junctional breakdown and vascular leakage (Orsenigo et al., 2012). Our study (Gordon et al., 2016) hinted for the first time that c-Src does not exclusively affect vascular permeability and barrier function (Eliceiri et al., 1999; Scheppke et al., 2008; Sun et al., 2012; Weis et al., 2004), but it also plays a role in sprouting angiogenesis (Gordon et al., 2016). In agreement with our observations, when all three SFKs (*Src*, *Yes* and *Fyn*) are constitutively knocked out, mice die at embryonic day 9.5, a time point that is characterized by active vasculogenesis. Similarly, viral delivery of a kinase-deleted c-Src results in decreased VEGF-induced angiogenesis in chicken chorioallantoic membrane (CAM) assays or when applied to mouse skin (Eliceiri et al., 1999). This suggests that SFK, including c-Src, may indeed be crucial for early vasculogenesis and possibly angiogenesis, but a confounding factor has consistently been that the other SFK members, such as Yes and Fyn, may be able to compensate for each other.

Here, we sought to identify the role for c-Src in sprouting angiogenesis using a conditional, inducible c-Src knockout mouse model. We report that c-Src is required for sprouting angiogenesis and vessel stability in *ex vivo* explants, and in the developing mouse trachea and retina via control of cell-matrix adhesion. In contrast, no major changes in VE-cadherin patterning or phosphorylation were observed upon loss of c-Src. Instead, we observed that central focal adhesion components paxillin and focal adhesion kinase (FAK) were phosphorylated downstream of c-Src in endothelial cells and in the sprouting front of the mouse retina. Taken together, our study reveals a novel role for c-Src in developmental angiogenic sprouting upstream of cell-matrix adhesion but not cell-cell adhesion, providing new insights for the importance of subcellular localization of intracellular kinases in regulating vascular adhesion and sprouting.

## RESULTS

### Endothelial c-Src is required for developmental angiogenesis

**Fig. 1.**
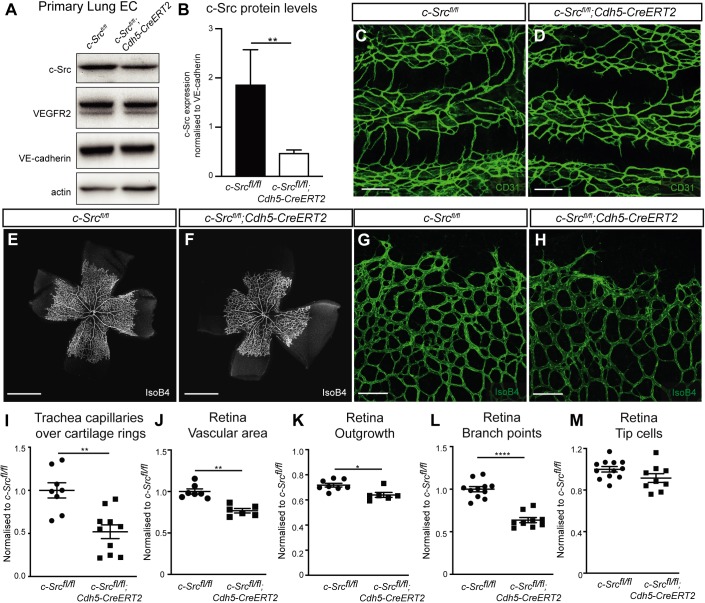
**c-Src is required for developmental angiogenesis.** (A) Endothelial cells were isolated from lungs of tamoxifen-treated *c-Src^flox/flox^* and *c-Src^flox/flox^; Cdh5-CreERT2* mice and c-Src protein knockdown was assessed by western blot. (B) A reduction in c-Src protein was observed in *c-Src^flox/flox^; Cdh5-CreERT2* mice compared with *c-Src^flox/flox^* (*n*>6). (C,D) Vasculature of tracheas from P6 mice immunostained for CD31. Scale bars: 100 µm. (E-H) Vasculature of retinas from P6 mice immunostained for isolectin B4. Scale bars: 1000 µm in E,F; 100 µm in G,H. (I) *Src*-deficient tracheas display reduced capillaries crossing cartilage rings of tracheas (*n*>8, where *n* is the number of mice). (J-M) *Src*-deficient retinas display reduced vascular area, reduced outgrowth from the optic nerve and a reduced number of branch points, but no change in the number of tip cells (*n*>6, where *n* is the number of retinas). *P<0.05, **P<0.01, ****P<0.0001. Data are mean±s.e.m. with individual data points indicated. Statistical significance was determined using a Mann–Whitney test.

Constitutive knockout of c-Src is reported to be compatible with grossly normal development (Soriano et al., 1991). However, renewed analysis of global c-Src-deficient mice showed postnatal defects in developmental angiogenesis of surviving mice, with moderate decreased vascular growth and increased vessel regression (Fig. S1). To specifically investigate the endothelial cell-autonomous role of c-Src, we generated mice with an inducible, endothelial-specific deletion of c-Src, by crossing c-Src-floxed mice (Fig. S2) with tamoxifen-inducible Cdh5CreERT2 mice (Kogata et al., 2006; Wang et al., 2010) (*c-Src^fl/fl^;Cdh5CreERT2*). When treated with tamoxifen after birth, these mice displayed a ∼75% reduction in c-Src expression in endothelial cells isolated from lungs compared with their wild-type counterparts (Fig. 1A,B). The deletion strategy (Lox sites placed between exons 7-9) could potentially result in expression of a truncated c-Src fragment; however, this was not detected (Fig. S2C). Therefore, our system is an endothelial cell c-Src loss-of-function model. Interestingly, expression of other SFKs, Yes and Fyn, also decreased upon loss of c-Src, although the effect was significant only for Fyn (Fig. S3A-C). These results suggest that compensation by overexpression of other SFKs did not occur as a consequence of temporal endothelial c-Src deficiency. The possibility that Fyn was turned over at a higher rate upon loss of endothelial c-Src needs further exploration.

In agreement with our findings from mice with a deletion of c-Src partner TSAd (*TSAd^fl/fl^;Cdh5CreERT2*; Gordon et al., 2016), *c-Src^fl/fl^;Cdh5CreERT2* mice displayed a decrease in capillary sprouting over the cartilage rings of the developing trachea at postnatal day (P) 5 compared with their wild-type littermates (Fig. 1C,D,I). Sensitivity of tracheal vessels to loss of c-Src is in agreement with results of Orsenigo et al. (2012), who observed an arrest in permeability upon treatment with a c-Src inhibitor. However, in contrast to mice with deletion of upstream signaling partners in the VEGFR2-949/TSAd/c-Src/VE-cadherin pathway (*TSAd^fl/fl^;Cdh5CreERT2* and *VEGFR2^Y949F/Y949F^* mice; Gordon et al., 2016; Li et al., 2016), c-Src-deficient mice displayed a significant reduction in the overall network density of the retinal vasculature (Fig. 1E-H). The total vascular area (Fig. 1J), outgrowth from the optic nerve (Fig. 1K) and number of branch points (Fig. 1L) were all reduced in *c-Src^fl/fl^;Cdh5CreERT2* mice compared with wild-type littermates. These differences in the requirement for VEGFR2-949/TSAd suggest that sprouting in the trachea and retina are controlled by unique mechanisms. In contrast, the number of tip cells in the retina was unaffected (Fig. 1M). When deletion was induced at P1-3 and retinas analyzed at P23, no changes in the vasculature in the deep or superficial plexus were observed, suggesting that a long-term deletion of c-Src is overcome possibly by compensatory mechanisms (Fig. S3D-L). In contrast, we did not observe any changes in a different vascular bed of the eye: the hyaloid plexus (Fig. S3M-P). These results show for the first time that c-Src is required for developmental angiogenesis in a time-dependent, and likely tissue-dependent, manner.

### Endothelial c-Src is required for vascular sprouting but not tip cell identity

**Fig. 2.**
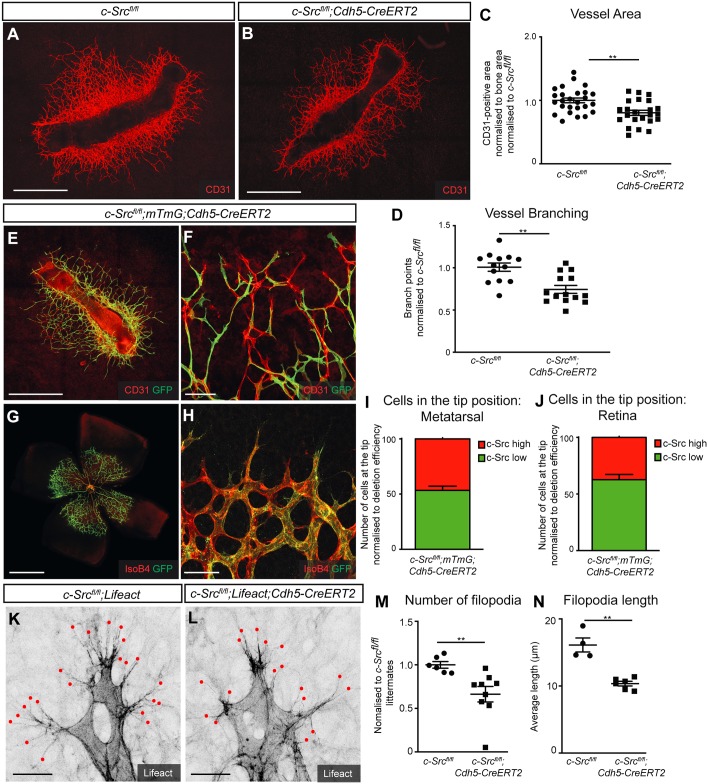
**c-Src regulates filopodial extension but not tip cell identity.** (A,B) Metatarsals from E16.5 embryos were cultured for 14 days and blood vessels immunostained for CD31. Scale bars: 1000 μm. (C,D) *Src*-deficient metatarsals displayed significantly reduced total vessel growth and branch points in vasculature from metatarsals (*n*>13, where *n* is the number of metatarsals). (E,F) *c-Src^flox/flox^; mTmG; Cdh5-CreERT2* metatarsals, where recombined cells express GFP and non-recombined cells are immunostained for CD31. Scale bars: 1000 µm in E; 100 µm in F. (G,H) *c-Src^flox/flox^; mTmG; Cdh5-CreERT2* retinas, where recombined cells express GFP and non-recombined cells are immunostained for isolectin B4. Scale bars: 1000 µm in G; 50 µm in H. (I,J) Quantification of recombined c-Src-deficient cells at the tip, normalized to overall contribution of cells to the endothelium. Statistical significance was determined by comparing the proportion of c-Src-deficient (green) cells at the tip with the total proportion of c-Src-deficient cells. No significant changes were observed. *n*=12, where *n* is the number of metatarsals (I) or retinas (J). (K,L) *c-Src^flox/flox^; Lifeact* and *c-Src^flox/flox^; Lifeact; Cdh5-CreERT2* retinas were analyzed for filopodial protrusions at the vascular front of P6 retinas. Red dots denote sites of filopodial termination. Scale bars: 25 µm. (M,N) c-Src-deficient retinas display reduced number of filopodia and average length of filopodia. The number of filopodia was quantified from a standardized length of the vascular front (1024 µm). *n*>4, *n* is number of retinas. **P<0.01. Data are mean±s.e.m. with individual data points indicated. Statistical significance was determined using a Mann–Whitney test.

To further investigate the requirement for c-Src in angiogenic sprouting, we used the mouse metatarsal assay to assess angiogenesis in an *ex vivo* setting (Song et al., 2015). In agreement with our findings in the trachea and retina, metatarsals isolated from *c-Src^fl/fl^;Cdh5CreERT2* mice displayed a significant decrease in angiogenesis (Fig. 2A,B), with a reduction in total vessel area (Fig. 2C) and branch points (Fig. 2D). This confirms the requirement for c-Src in angiogenic sprouting *in vivo* and *ex vivo.*

During sprouting angiogenesis, sprouts comprise highly migratory ‘tip’ cells with protruding filopodia, followed by their neighboring ‘stalk’ cells, which form the stable lumenized sprout (Gerhardt et al., 2003). To assess the cell-autonomous requirement of c-Src in tip cell identity, we crossed mice onto an mTmG reporter background (Muzumdar et al., 2007) (*c-Src^fl/fl^;mTmG;Cdh5CreERT2*) and assessed the relative contribution of GFP-positive, c-Src-deficient cells to the tip region in chimeric settings [as described previously (Aspalter et al., 2015)]. In agreement with the intact number of tip cells in c-Src-deficient retinas, we observed no requirement for c-Src in the formation of tip cells in metatarsals (Fig. 2E,F,I) or in the retina (Fig. 2G,H,J). Thus, despite reduced sprouting in both of these assays, cells deficient in c-Src were still able to reach the tip position. In agreement, c-Src activity is not as prominent in leading cells as it is in trailing stalk cells when assessed using a HUVEC scratch assay, indicating that the placement in the leading position is not strictly dependent on c-Src function (Fig. S4). Interestingly, when mice were crossed with *Lifeact-EGFP* mice, to allow for high resolution visualization of filopodia extending from tip cells and where endothelial cells display strong GFP intensity (Fraccaroli et al., 2012; Riedl et al., 2010), we observed fewer and significantly shorter filopodia in tip cells of *c-Src^fl/fl^;Lifeact;Cdh5CreERT2* retinas compared with wild-type *Lifeact;Cdh5CreERT2* retinas (Fig. 2K-N). EGFP-positive vessels were co-stained with IsolectinB4 and VE-cadherin to confirm endothelial identity (Fig. S5). This suggests that c-Src acts to promote angiogenesis independently of tip cell selection, yet is required for the proper function of tip cells by regulating filopodia formation and extension to drive the growth of sprouting vessels.

### Reduced vascular density in c-Src-deficient retinas is the result of decreased vessel stability

**Fig. 3.**
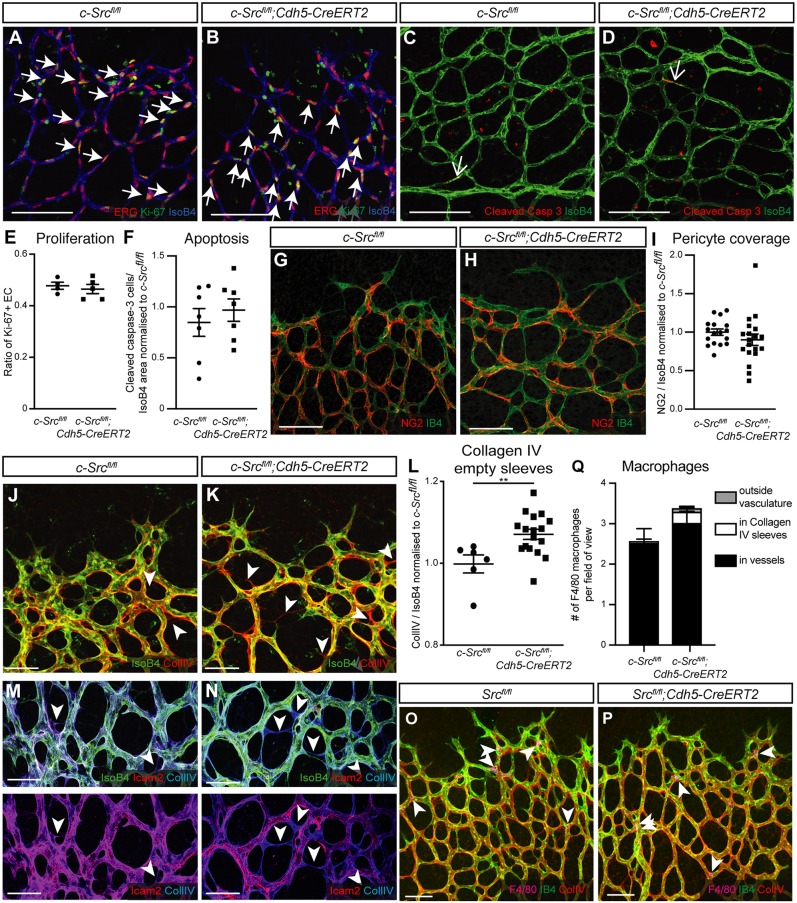
**c-Src controls vascular density through vessel stability.** (A,B) Proliferating cells in *c-Src^flox/flox^* and *c-Src^flox/flox^; Cdh5-CreERT2* retinas at P5 were detected by Ki67 immunostaining. Arrows indicate Ki67-positive cells. Scale bars: 100 µm. (C,D) Apoptosis in *c-Src^flox/flox^* and *c-Src^flox/flox^; Cdh5-CreERT2* retinas at P5 was detected by cleaved caspase 3 immunostaining. Arrows indicate apoptotic cells. Scale bars: 100 µm. (E) No changes were detected in endothelial cell proliferation upon loss of c-Src (*n*>4, where *n* is the number of retinas). (F) No changes were detected in endothelial cell apoptosis upon loss of c-Src (*n*>4, where *n* is the number of retinas). (G,H) Pericyte coverage in *c-Src^flox/flox^* and *c-Src^flox/flox^; Cdh5-CreERT2* retinas at P5 was assessed by NG2 immunostaining. Scale bars: 100 µm. (I) No changes were detected in pericyte coverage upon loss of c-Src (*n*>4, where *n* is the number of retinas). (J,K) Visualization of empty collagen IV sleeves in *c-Src^flox/flox^* and *c-Src^flox/flox^; Cdh5-CreERT2* retinas at P5. Arrowheads indicate empty sleeves in the retinal vasculature. Scale bars: 50 µm. (L) *c-Src^flox/flox^; Cdh5-CreERT2* retinas at P5 display an increased number of empty collagen IV sleeves in the retinal vasculature compared with *c-Src^flox/flox^* littermates (*n*>6, where *n* is the number of retinas). (M,N) Empty collagen IV sleeves in both *c-Src^flox/flox^* and *c-Src^flox/flox^; Cdh5-CreERT2* retinas lack Icam2 expression, suggesting c-Src is not required for initial vessel lumen formation, but for lumen maintenance and stability. Arrowheads indicate empty collagen IV sleeves. Scale bars: 50 µm. (O,P) Visualization of macrophages by F4/80 immunostaining in *c-Src^flox/flox^* and *c-Src^flox/flox^; Cdh5-CreERT2* retinas. Arrowheads indicate macrophages. Scale bars: 50 µm. (Q) *c-Src^flox/flox^; Cdh5-CreERT2* retinas at P6 display no difference in the number of macrophages within the vasculature or associated with empty collagen IV sleeves compared with *c-Src^flox/flox^* littermates (*n*>6, where *n* is the number of retinas). **P<0.01. Data are mean±s.e.m. with individual data points indicated. Statistical significance was determined using a Mann–Whitney test.

As we observed a reduction in total vascular area and branch points in *c-Src^fl/fl^;Cdh5CreERT2* retinas (Fig. 1J,L), we reasoned this may be due to changes in cell proliferation, cell death or vascular regression. In *c-Src^fl/fl^;Cdh5CreERT2* retinas, we found no change in Ki-67-positive, proliferating cells (Fig. 3A,B,E) or in cleaved caspase 3 cells undergoing apoptosis in the vascular plexus (Fig. 3C,D,F), indicating c-Src does not control endothelial cell proliferation or cell death. We also did not observe any changes in the degree of pericyte coverage of the vasculature in *c-Src^fl/fl^;Cdh5CreERT2* retinas (Fig. 3G-I).

To study vessel regression, we performed immunostaining with type IV collagen (CollIV), which allows for visualization of regressed vessels by empty matrix sleeves (Franco et al., 2015). We observed a significant increase in the number of empty CollIV sleeves in the retinal vasculature of *c-Src^fl/fl^;Cdh5CreERT2* mice (Fig. 3J-L), occurring independently of changes in apoptosis (Fig. 3F). As c-Src and Fyn have been shown to be crucial for the lumenization of the vasculature (Koh et al., 2009), we performed immunostaining for intercellular adhesion molecule 2 (ICAM2), which marks the apical/luminal endothelial cell membrane (Stenzel et al., 2011). We observed ICAM2 staining was lost where empty matrix sleeves were observed (Fig. 3M,N), as reported previously (Franco et al., 2015). However, the existing vasculature was able to form a lumen, suggesting c-Src is not required for the initial lumenization of the vasculature. We also did not observe any changes in the number or association of macrophages with the vasculature or with CollIV sleeves upon loss of c-Src (Fig. 3O-Q). This is pertinent, given the known role of c-Src in osteoclasts, the role of macrophages in regression and the recent data demonstrating *Cdh5CreERT2* mice can display non-endothelial Cre activity (He et al., 2019). Taken together, these results suggest that, although c-Src is not required for the formation of the vascular lumen, it is required for the stability of newly formed vessels.

### VE-cadherin phosphorylation is not controlled by c-Src at the sprouting front of the retina

We hypothesized that the requirement for c-Src to stabilize the growing vascular network was due to altered VE-cadherin phosphorylation at two tyrosine residues that have been shown to be important for its internalization, Tyr658 and Tyr685 (Orsenigo et al., 2012; Wessel et al., 2014). If VE-cadherin phosphorylation and junctional distribution were altered, we would predict the inability of sprouts to form stable connections with their neighbors, resulting in decreased vascular stability.

**Fig. 4.**
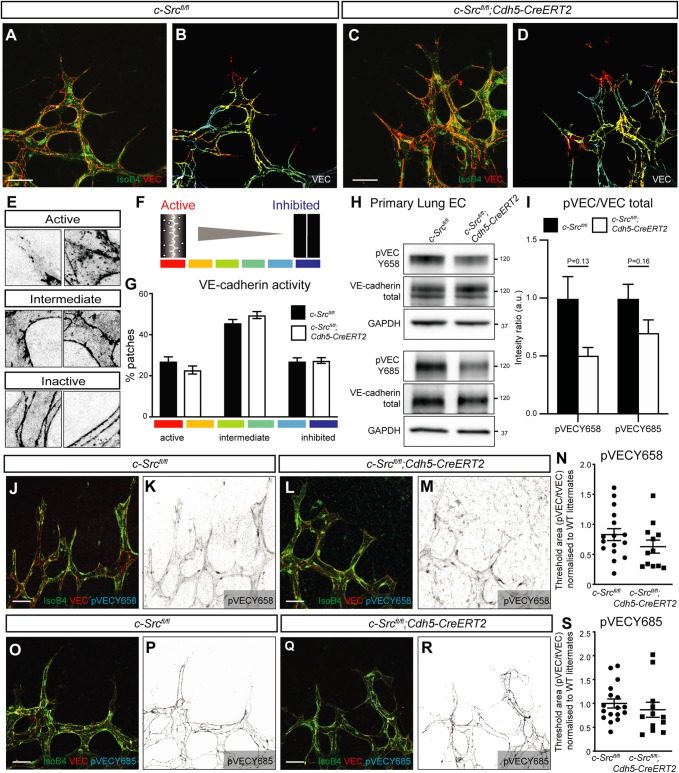
**VEGFA-induced c-Src activation does not significantly alter VE-cadherin patterning.** (A-D) High-magnification images of the sprouting front of retinas. B and D are heat maps of the VE-cadherin (VEC) morphology of retinas in A and C, respectively. Scale bars: 25 µm. (E,F) Representative patches of VE-cadherin morphology, from active (warm colors) to intermediate to inactive (cool colors). (G) Quantification of A-D. The VE-cadherin morphology in each patch was manually classified using a scale from active (red, serrated line and bright interior) to inactive (blue, straight line and dark interior). *n*=4 (where *n* is the number of retinas, >150 patches quantified per retina). (H) Endothelial cells were isolated from lungs of *c-Src^flox/flox^* and *c-Src^flox/flox^; Cdh5-CreERT2* mice, and protein expression was assessed by western blot. (I) No significant decrease in p-VE-cadherin at either Y658 or Y685 was observed by western blot (*n*>6 mice). (J-M) High-magnification images of the sprouting front of retinas immunostained for VE-cadherin and phospho-VE-cadherin Y658 (pVECY658). (K,M) Single-channel images of pVECY658 in J,L, respectively. Scale bars: 25 µm. (N) Quantification of pVECY658 staining area normalized to total VEC immunostaining (*n*>12, where *n* is the number of retinas). (O-R) High-magnification images of the sprouting front of retinas immunostained for VE-cadherin and phospho-VE-cadherin Y685 (pVECY685). Scale bars: 25 µm. (P,R) Single-channel images of pVECY685 in O,Q, respectively. (S) Quantification of pVECY685 staining area normalized to total VEC immunostaining. (*n*>12, where *n* is the number of retinas). Data are mean±s.e.m. with individual data points indicated. Statistical significance was determined using a Mann–Whitney test.

To characterize cell-cell junctions in the c-Src-deleted retina, a number of analyses were performed. Based on immunostaining for erythrocytes, the vascular barrier in the c-Src-deficient retinas appeared unaffected as there was no apparent hemorrhage (Fig. S6). Moreover, high resolution confocal imaging analyses (Bentley et al., 2014) were used to define the degree of adherens junction stability (Fig. 4A-G). Regions of the retina displaying empty ColllV sleeves due to loss of c-Src expression were selected for these analyses (Fig. S7). Here, the pattern of segmented VE-cadherin junctions was classified on a gradient between ‘active’ (serrated and vesicular appearance) or ‘inactive’ (straighter appearance with fewer vesicles) (Fig. 4E,F). Unexpectedly, no changes in VE-cadherin morphology were observed between wild-type and c-Src-deficient vessels at the sprouting front of the retina (Fig. 4G), suggesting vessel stability may not be regulated by a c-Src-VE-cadherin phosphorylation cascade. Analysis of phosphorylated VE-cadherin from endothelial cells isolated from lungs also did not reveal significant changes in phosphorylation at Tyr 658 or Tyr685 (Fig. 4H,I). These results were confirmed by immunostaining for phosphorylated VE-cadherin Tyr658 (Fig. 4J-N) and VE-cadherin Tyr685 (Fig. 4O-S), both of which showed no significant change in intensity at VE-cadherin-positive cell junctions in the postnatal retina vasculature upon loss of c-Src. Our results suggest that the loss of vascular density and stability upon loss of c-Src in the retina is independent of VE-cadherin phosphorylation and cell-cell adhesion.

### c-Src is required for controlled directional sprout movement

To further investigate the dynamics of vessel growth upon loss of c-Src, we used the mouse metatarsal assay to visualize the sprouting vasculature in an *ex vivo* setting. We hypothesized that if reduced sprouting upon loss of c-Src was independent of altered VE-cadherin junctional localization and cell-cell adhesion, then the dynamics of sprout elongation or sprout stability could instead be dependent on altered cell-matrix regulation.

**Fig. 5.**
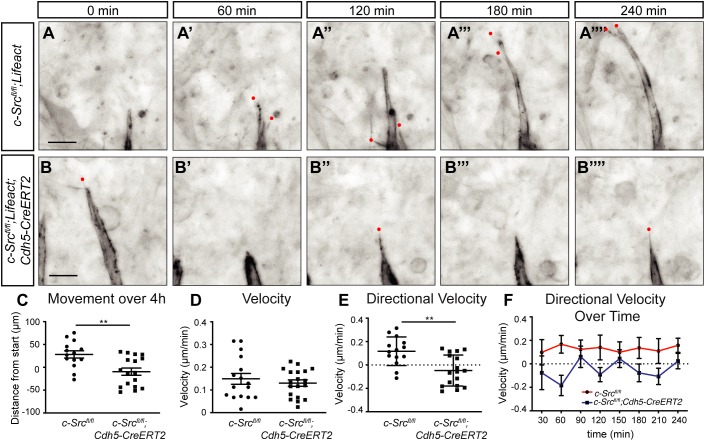
**c-Src is required for vessel growth but not for the velocity of sprout movement.** (A-B″″) Metatarsals from *c-Src^flox/flox^; Lifeact* and *c-Src^flox/flox^; Lifeact; Cdh5-CreERT2* E16.6 embryos were imaged over 4 h at 37°C. An image for every hour is shown. Red dots indicate filopodia. Scale bars: 25 µm. (C) c-Src-deficient sprouts displayed reduced forward movement. (D) Velocity of total sprout movement over 4 h. c-Src-deficient sprouts displayed no changes in overall sprout velocity. (E) Directional velocity of total sprout movement over 4 h. Positive values are forward movement, negative values are backwards movement. c-Src-deficient sprouts displayed reduced forward sprout velocity. (F) Directional velocity over 30-min increments. c-Src-deficient sprouts had both forward and backwards movement, demonstrating erratic movements. Wild-type sprouts moved in a largely consistent forward direction (*n*>14, where *n* is number of sprouts from three independent experiments). **P<0.01. Data are mean±s.e.m. with individual data points indicated. Statistical significance was determined using a Mann–Whitney test.

Using *Lifeact-EGFP* metatarsals enabled us to clearly view sprouting speed, filopodial extensions and directional growth of the vasculature at high resolution. Vascular structures were shown to express CD31, confirming endothelial identity (Fig. S5). Similar to what we observed in the retina, fewer filopodial extensions were visualized in sprouts deficient for c-Src (Fig. 5A,B). Live imaging enabled us to visualize a highly unstable sprouting profile of vessels upon loss of c-Src. Although sprouts were still able to move forwards (Fig. 5B′-B‴), when movement over a 4 h time period was quantified, we observed a significant decrease in the total distance of growth in *c-Src^fl/fl^;Lifeact;Cdh5CreERT2* metatarsals compared with *c-Src^fl/fl^;Lifeact* (Fig. 5C). The total velocity, including both forward or backward movement of the sprout, was unchanged upon loss of c-Src (Fig. 5D). However, compared with wild type, c-Src-deficient sprouts often moved in a reverse direction (negative velocity from point to point) (Fig. 5E), resulting in their net decreased forward movement (Fig. 5C). When measured over 30 min increments, c-Src-deficient sprouts moved both forwards and backwards, in contrast to wild-type sprouts, which moved in a consistent forward manner (Fig. 5F). This reveals that c-Src mediates controlled directional movement during angiogenesis and that c-Src-deficient sprouts are highly unstable.

### Phosphorylation of c-Src can occur independently of VEGFA/VEGFR2 signaling

In order to further investigate the pathways that may be up or downstream of c-Src to control vascular stability in retinal angiogenesis, we manipulated c-Src levels by knockdown using siRNA or overexpression by transducing endothelial cells using lentiviral transfection of an mKate2-tagged c-Src. So as not to interfere with c-Src protein folding or activity, the mKate2 tag was attached to the full-length protein using a glycine/serine-rich flexible linker peptide (as described by Sandilands et al., 2004). Addition of this linker/tag does not interfere with c-Src phosphorylation activity or its binding to interaction partners (Sandilands et al., 2004).

**Fig. 6.**
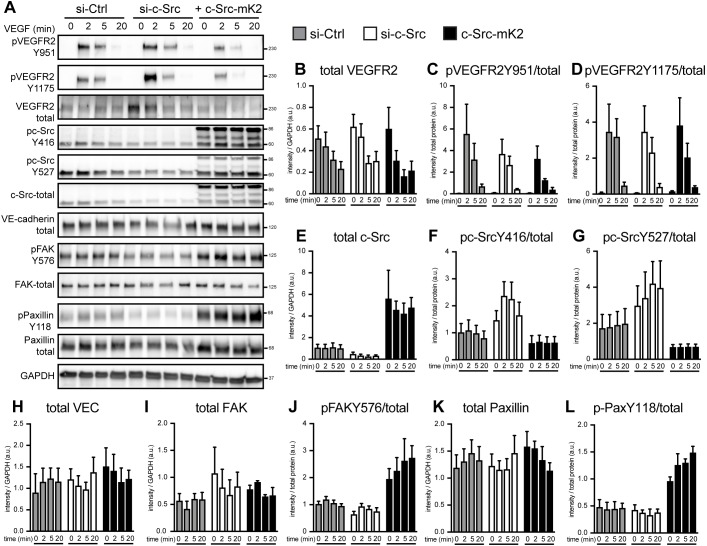
**VEGFA/VEGFR2 activation does not alter c-Src phosphorylation.** (A) HUVECs transfected with Control or c-Src siRNA, or with overexpression of c-Src-mKate2 were treated with VEGFA for 0, 2, 5 or 20 min. Activation of proteins was assessed using phospho-specific antibodies and western blotting. (B-D) Total VEGFR2 levels (normalized to GAPDH loading control) decreased upon activation by VEGFA, shown by phosphorylation on Y951 and Y1175 (normalized to total VEGFR2 expression). (E-G) Upon VEGFA stimulation, c-Src did not become phosphorylated at Y416 or Y527 (normalized to total c-Src expression). (H) Total VE-cadherin levels (normalized to GAPDH loading control) did not significantly change upon stimulation with VEGFA, c-Src depletion or overexpression. (I,J) Total FAK levels (normalized to GAPDH loading control) and activated Y576 FAK (normalized to total FAK) are not responsive to VEGFA stimulation. c-Src-mK2 overexpression increased FAK activation at Y576. (K,L) Total paxillin levels (normalized to GAPDH loading control) and activated Y118 paxillin, did not increase with VEGFA stimulation. c-Src-mK2 overexpression increased paxillin activation at Y118. *n*=3 from three independent experiments. Data are mean±s.e.m.

When we stimulated these c-Src-manipulated endothelial cells with VEGFA, total VEGFR2 levels decreased as a consequence of ligand-induced internalization at similar rates across samples (Fig. 6A,B) and VEGFR2 phosphorylation at Tyr951 (associated with TSAd binding and c-Src activity at cell junctions) or Tyr1175 were downregulated (Fig. 6A,C,D). These data indicate that c-Src does not alter the rate of VEGFR2 phosphorylation, internalization or degradation, and suggest that c-Src is not upstream of VEGFR2 signaling when HUVECs are grown on fibronectin. c-Src expression was three times lower when cells were treated with siRNA, and when treated with c-Src-mKate2 lentivirus, we observed a 4.5-fold increase in c-Src protein (Fig. 6A,E). Of note, in c-Src-silenced cells, the remaining pool of c-Src was phosphorylated with increased stoichiometry (pc-Src/total c-Src) both at Tyr416 and Tyr527, suggesting compensation for the reduced c-Src protein levels (Fig. 6A,F,G). Moreover, in agreement with Fig. 4, VE-cadherin total protein was unchanged after c-Src manipulation, suggesting c-Src is acting independently of VE-cadherin phosphorylation, internalization and degradation/recycling (Fig. 6A,H).

Although total protein levels of focal adhesion components FAK and Paxillin were unchanged upon c-Src manipulations (Fig. 6A,I,K), phosphorylation of FAK at one of the c-Src target sites (Jean et al., 2014; Ma et al., 2019), Tyr576 (Fig. 6A,J), and phosphorylation of paxillin at Tyr118 were significantly increased upon c-Src-mKate2 overexpression (Fig. 6A,L). These changes were not altered upon VEGFA simulation, suggesting c-Src regulates focal adhesion component phosphorylation independently of VEGFA activation.

### Focal adhesion activation requires c-Src during retinal angiogenesis

**Fig. 7.**
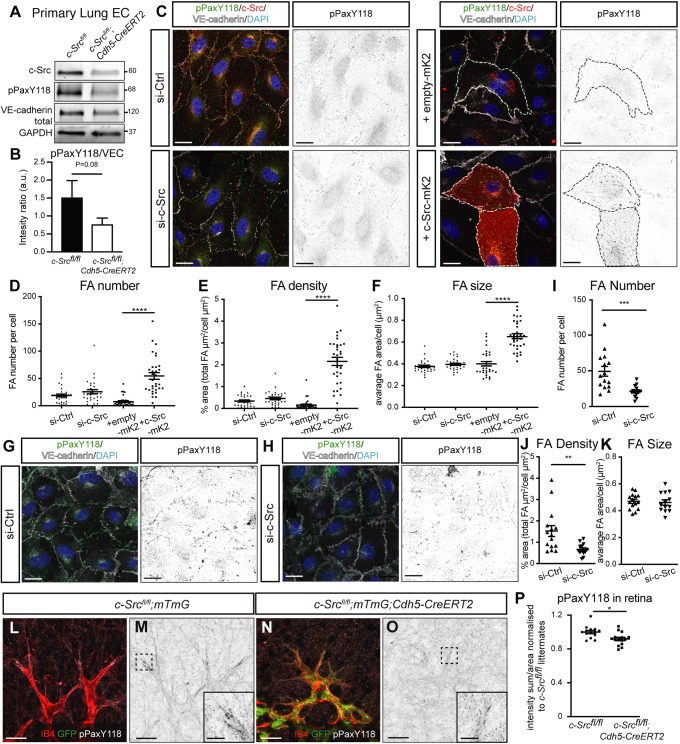
**c-Src mediates paxillin phosphorylation and subsequent focal adhesion formation.** (A) Endothelial cells were isolated from lungs of *c-Src^flox/flox^* and *c-Src^flox/flox^; Cdh5-CreERT2* mice, and protein expression was assessed by western blot. (B) A decrease in phospho-paxillin at Y118 was observed (*n*>5 mice). (C) HUVECs transfected with siRNA for control or c-Src, or with overexpression of empty-mKate2 or c-Src-mKate2 cultured on fibronectin-coated coverslips were immunostained for p-paxillin-Y118 (pPaxY118), c-Src (for siRNA-treated cells), VE-cadherin and DAPI. c-Src-mKate2 is visualized with mKate2 signal. Single channel images of pPaxY118 are shown on the right; mKate2-positive cells are outlined with dashed lines. Scale bars: 20 µm. (D-F) Focal adhesion number, density and size based on pPaxY118 staining was significantly increased upon c-Src-mKate2 overexpression (*n*>30 cells in total from three independent experiments). (G,H) HUVECs transfected with siRNA for control or c-Src cultured on vitronectin-coated coverslips were stained for p-paxillin-Y118 (pPaxY118), VE-cadherin and DAPI. Single channel images of pPaxY118 are shown on the right. Scale bars: 20 µm. (I-K) Focal adhesion number and density based on pPaxY118 staining was significantly decreased upon c-Src depletion, while the size was not significantly changed. *n*>15 cells in total from three independent experiments. (L-O) *c-Src^flox/flox^; mTmG* and *c-Src^flox/flox^; mTmG; Cdh5-CreERT2* retinas were immunostained with pPaxY118. (M,O) Single channel pPaxY118. Insets show higher magnification of the sprouting front in the boxed area. Scale bars: 25 µm; 5 µm in insets. (P) A significant decrease in the pPaxY118 staining intensity of the vasculature (normalized to pPaxY118 staining of the non-vascular retina) was observed upon loss of c-Src. *n*>15, where *n* is the number of retinas. *P<0.05, **P<0.01, ***P<0.005, ****P<0.001. Data are mean±s.e.m. with individual data points indicated. Statistical significance was determined using a Wilcoxon matched-pairs signed rank test (D-F) or a Mann–Whitney test (B,I-K,P).

To further investigate the regulation of focal adhesion components in mediating c-Src-dependent vessel stability, we closely examined paxillin phosphorylation in endothelial cells isolated from mouse lungs. We initially confirmed c-Src expression is significantly reduced in cells isolated from *c-Src^fl/fl^;Cdh5CreERT2* mice (Fig. 1A) and subsequently examined phosphorylation of paxillin at Tyr118. We saw a trending decrease of paxillin phosphorylation (*P*=0.08), in line with the hypothesis that c-Src mediates vascular stability in endothelial cells through cell-matrix adhesion assembly (Fig. 7A,B). We also observed enriched phospho-c-Src immunostaining in focal adhesions in a scratch assay (Fig. S4C). However, phospho-c-Src was also enriched at cell-cell junctions in a monolayer (Fig. S4D), again confirming previous studies showing that c-Src controls adherens junction activity (Gordon et al., 2016; Orsenigo et al., 2012), although in a context-dependent manner.

To examine the effect of focal adhesion size and distribution in endothelial cells with c-Src knockdown or overexpression, we again treated cells with c-Src siRNA or c-Src-mKate2 lentivirus, grew cells on fibronectin (thought to engage integrin α5β1) (Schaffner et al., 2013) and immunostained to detect phospho-paxillin Tyr118 (Fig. 7C). A reduction of c-Src in cultured endothelial cells grown on fibronectin did not have an impact on focal adhesion number (Fig. 7D), density (Fig. 7E) or size (Fig. 7F), despite a significant reduction in the intensity of c-Src protein by immunostaining (Fig. S8A). However, overexpression of c-Src resulted in an increase in number, density and size of FAs compared with cells transfected with empty-mKate2 lentivirus (Fig. 7C-F). The lack of change in FA upon c-Src knockdown may be due to increased phosphorylation of the remaining pool of c-Src (Fig. 6), or may be due to cells being grown on fibronectin matrix. In ECs, individual matrix components induce focal adhesions by clustering of their individual receptors (Dejana et al., 1988). Alteration of fibronectin fibrillogenesis is known to be mediated through paxillin phosphorylation (Zaidel-Bar et al., 2007); however, fibronectin deposition was unaffected by the loss of c-Src (Fig. S9). To investigate whether other matrix components may regulate how FAs are controlled by c-Src, we grew c-Src-deficient cells on vitronectin, known to preferentially engage integrin αvβ3 (Felding-Habermann and Cheresh, 1993; Horton, 1997), which resulted in decreased FA number and density (Fig. 7G-K). This confirms that c-Src controls focal adhesion activity and cell-matrix connections in endothelial cells, potentially by engaging integrin αvβ3.

The existence of focal adhesion-type structures *in vivo* is still under considerable debate, but it is known that focal adhesion-like signaling pathways do exist *in vivo* in the retina (Raimondi et al., 2014). Therefore, we sought to examine differential paxillin phosphorylation in the retina of c-Src-deficient mice. We observed that the intensity of phospho-paxillin Tyr118 staining was the brightest at the leading edge of the retina, and enriched where filopodia extend from tip cells (Fig. 7L,M,P). In c-Src-deficient endothelial cells of the retina, this intensity of staining was significantly reduced (Fig. 7N,O,P). This reveals that the inability of c-Src-deficient endothelial cells to form a stable cell-matrix connection in the sprouting retina causes the reduced vascular density and increased vascular regression in these mice.

## DISCUSSION

c-Src is a widely expressed non-receptor tyrosine kinase with crucial functions in a multitude of cell types (Espada and Martín-Pérez, 2017). Global deletion of c-Src in mice results in osteopetrosis due to impaired osteoclast function (Soriano et al., 1991), and to decreased tumor angiogenesis and vascular permeability (Eliceiri et al., 1999); however, potential effects on developmental angiogenesis have not been explored. Here, we show that c-Src is important for postnatal vascular development in the retina and trachea, specifically by regulating endothelial cell-matrix adhesion, and is crucial for the outward growth and stabilization of new vessels during sprouting angiogenesis. In contrast, cell-cell adhesion during developmental angiogenesis appeared unaffected. We also found that the main effect of c-Src deficiency was in decreased filopodial stability and reduced postnatal outgrowth and branching within the vascular plexus of the retina, accompanied by a loss of paxillin phosphorylation at the vascular front of the plexus.

One possible reason we did not observe changes in adherens junction activity, but did find reduced focal adhesion activation upon loss of c-Src in the developing retina, is the unique properties of the endothelial barrier. In previous studies specifically deleting the adaptor molecule TSAd from endothelial cells (Gordon et al., 2016), we have previously shown that the VEGFR2-TSAd-c-Src-VE-cadherin signaling complex is required for sprouting angiogenesis in the tracheal vasculature, but not in the developing retina. While TSAd binds to the phosphosite pY949 in VEGFR2, c-Src may have more extensive, non-VEGF-dependent effects in the retina. Whether genetic loss of c-Src affects adherens junctions or FAs in settings of high VEGF exposure (such as oxygen-induced retinopathy) or across different tissue beds is the focus of ongoing work.

Phosphorylation of VE-cadherin, in particular on Tyr685, is dependent on VEGFA and correlates with increased vascular permeability *in vivo* (Orsenigo et al., 2012; Wessel et al., 2014). Accordingly, pharmacological c-Src blockade or constitutive gene targeting of c-Src reduces paracellular permeability (Eliceiri et al., 1999; Paul et al., 2001). Treatment with a c-Src inhibitor results in loss of VE-cadherin-dependent permeability, providing further evidence that tracheal vessels are sensitive to c-Src-mediated VE-cadherin phosphorylation (Orsenigo et al., 2012). Although we did not find changes in phospho-VE-cadherin levels or in the arrangement of adherens junctions within this study, we have not investigated whether loss of c-Src results in alterations in vascular permeability in the retina.

Potential changes in permeability may be independent of VE-cadherin phosphorylation, which would be in contrast to the well-documented pattern in non-CNS organs (Orsenigo et al., 2012). Indeed, the blood-retinal barrier is dependent on adherens junction stability, but also on regulation of endothelial transcytosis (Chow and Gu, 2017), which is mediated by caveolae. Caveolin 1 (Cav1), a known c-Src substrate (Zimnicka et al., 2016), is essential for caveolae formation (Drab et al., 2001; Razani et al., 2001). However, the exact role of Cav1 (and caveolae) in transcytosis has not been determined. Although albumin transport across the endothelium requires Cav1 and caveolae, animals lacking Cav1 display increased albumin leakage in the retinal vasculature (Gu et al., 2014). Additionally, recent studies have shown no role for Cav1 and caveolae in transport across the endothelium in tumors (Sindhwani et al., 2020). Conclusively discerning the effects of c-Src deletion on endothelial transcytosis and the blood-retinal barrier cannot be achieved using confocal microscopy; instead, electron microscopy must be employed.

The interpretation of previous data on the role of c-Src in endothelial cell biology, whether *in vitro* or *in vivo*, is complicated by the possibility that the related EC-enriched SFKs Yes and Fyn may have been affected as a result of compensation. Although c-Src, Yes and Fyn are structurally highly related and are ubiquitously expressed, it is increasingly appreciated that they play distinct roles in the endothelium. However, their role in angiogenesis remains to be addressed using appropriate genetic models. Here, we show unexpected biology as a result of deleting c-Src in endothelial cells that are not compromised by compensatory overexpression of Yes and Fyn. In contrast, deletion of c-Src also resulted in a significant loss of Fyn expression, possibly owing to Fyn being biologically active and turned over at a higher rate, in order to compensate for loss of c-Src. Fyn has been implicated in signaling downstream of VEGFR2 in endothelial cells, specifically involving Tyr1212 in VEGFR2 (Lamalice et al., 2006). However, these results from *in vitro* models were not recapitulated when examining a VEGFR2 mutant with a Tyr to Phe exchange at the 1212 position (Testini et al., 2019). Fyn has also been implicated in lumen formation in tissue-cultured endothelial cells (Kim et al., 2017). Of note, lumen formation proceeded normally in the c-Src-deficient retinal vasculature (Fig. 3M,N). The *in vivo* role of Fyn remains to be studied using conditional endothelial cell-specific knockout models.

Although focal adhesions are known to control cell migration, precisely how they mediate endothelial cell behavior, function and formation of a functional vasculature, is a topic of intense study. The morphology and dynamics of FAs in cultured endothelial cells are controlled by the extracellular matrix and expression pattern of integrins, but are also influenced by different stimuli, such as VEGFA (Mui et al., 2016). *In vivo*, FAs can also be regulated by hemodynamic forces (Collins et al., 2014). c-Src is a substrate for FAK activity in FAs *in vitro* and is a component of the mechanosensory complex in endothelial cells (Tzima et al., 2005). However, c-Src can also act upstream of FAK, indicating the complexity of interactions between these kinases at FAs (Jean et al., 2014; Kleinschmidt and Schlaepfer, 2017). Expression of a kinase-dead form of FAK results in embryonic lethality at E9.5, which is associated with defects in the formation of the embryonic vasculature (Lim et al., 2010) as a result of impaired FA turnover and cell motility. Interestingly, in MEFs from these mice, FAK was placed upstream of c-Src, with kinase-dead FAK resulting in a loss of c-Src phosphorylation at its activating Tyr416 (Lim et al., 2010). Our data here meanwhile suggest c-Src acts upstream of FAK in ECs, as we observed increased FAK phosphorylation upon induction of c-Src expression (Fig. 6). Furthermore, based on the *in vivo* results presented here, we can place paxillin downstream of c-Src.

Although the exact sequence of events is not clear, as detailed above, activation of integrins triggers the assembly of the multi-protein FAK/c-Src/paxillin/talin complex linked to the cytoskeleton at nascent adhesions (Kleinschmidt and Schlaepfer, 2017). In cultured endothelial cells, c-Src promotes FAK phosphorylation at Tyr861 and association with αvβ5 upon VEGF stimulation (Eliceiri et al., 2002). αvβ5 is also required for angiogenesis (Friedlander et al., 1995); therefore, it is possible that c-Src activation downstream of this FAK-integrin complex may control FA assembly and vascular stability in the retina. β1 Integrin is also associated with angiogenesis through correct positioning of VE-cadherin (Pulous et al., 2019; Yamamoto et al., 2015). Given the lack of detectable VE-cadherin abnormalities in c-Src-deficient retinas and the lack of changes in focal adhesions upon loss of c-Src when cells are coated on fibronectin (Fig. 7), we predict that our phenotype is unlikely to be downstream of β1 Integrin activation. Our results culturing c-Src-deficient cells on vitronectin are suggestive of a role for αvβ3, which is associated with angiogenesis. However, this effect is context dependent (Demircioglu and Hodivala-Dilke, 2016; Plow et al., 2014) and vitronectin is known to engage other integrins (Felding-Habermann and Cheresh, 1993); therefore, although our results are suggestive, they are not yet conclusive. Future work aims to precisely decipher the time-dependent upstream (integrin) and downstream (FAK, talin, cytoskeleton) effectors of c-Src in sprouting angiogenesis.

Given the reported findings that both c-Src and FAK modulate VE-cadherin phosphorylation, and the findings here that c-Src regulates both FAK and FAs, a key question is whether there is coordination between cell-cell and cell-matrix adhesions in the regulation of angiogenesis and vascular permeability, and whether this is under the control of the same signals. Close interactions between integrins and cadherins have been associated with junctional stability (Chattopadhyay et al., 2003; Pulous et al., 2019; Weber et al., 2011; Yamamoto et al., 2015), and a means of direct communication between these compartments could moreover involve direct interaction between FAK and VE-cadherin. This has been shown to be crucial in the regulation of paracellular permeability in a manner independent of c-Src but dependent on FAK-mediated phosphorylation of β-catenin, independently of tension (Chen et al., 2012). Studies with a kinase-dead form of FAK revealed that FAK controls VEGF-induced VE-cadherin phosphorylation at Tyr658, with c-Src implicated upstream of FAK in this process (Jean et al., 2014). Inhibition of FAK resulted in reduced c-Src at cell-cell junctions; therefore, whether c-Src and FAK act in parallel or separately to regulate adherens junctions remains unsettled. To conclude, the results here indicate that cell-cell and cell-matrix adhesion *in vivo* can be separately controlled by c-Src; however, we do not exclude the possibility that there may be tissue and developmental stages when coordination between the two pathways is essential.

In summary, our results are the first to tie c-Src, a known mediator of vascular permeability, to sprouting angiogenesis. Although the dynamics of the assembly of the multi-protein FA complex and how this is regulated by c-Src remain to be fully elucidated, we observed that c-Src primarily controls cell-matrix adhesion through phosphorylation of the FA component paxillin. As c-Src inhibitors are already used in clinical settings, our results raise the possibility of improved tailoring of such treatment by providing a fundamentally deeper understanding of the role of c-Src in endothelial cell signaling, and how regulation of cell-cell and cell-matrix adhesion has a bearing on sprouting angiogenesis.

## MATERIALS AND METHODS

### Antibodies

The following antibodies were used: rat anti-CD31 (BD Biosciences, 553370; RRID, AB_394816; EC isolation, 250 µg/mouse), rat anti-ICAM2 (BD Biosciences, 553326; RRID, AB_394784; immunohistochemistry, 1/200), rabbit anti-phospho VE-cadherin (Tyr^658^ and Tyr^685^) [from E. Dejana (Orsenigo et al., 2012); western blot, 1/500; immunohistochemistry, 1/50], rabbit anti-phospho VE-cadherin (Tyr^658^) (Invitrogen, 44-1144G; RRID, AB_2533583; western blot, 1/1000), rabbit anti-GFP (Santa Cruz, sc8334; RRID, AB_641123; immunohistochemistry, 1/200), rat anti-VE-cadherin (BD Bioscience, 555289; RRID, AB_395707; immunohistochemistry, 1/100), goat anti-VE-cadherin (R&D Systems, AF1002, RRID, AB_2077789; western blot, 1/1000), isolectin-B4 directly conjugated to Alexa 488, 568 and 647 (Life Technologies/ThermoFisher Scientific, I21411, I21412 and I32450; RRID, AB_2314662; immunohistochemistry, 1/500), rabbit anti-phosho paxillin (Tyr^118^) (Abcam, AB4833; RRID, AB_304669; immunohistochemistry, 1/100), mouse anti-c-Src GD11 (Millipore, 05-184; RRID, AB_2302631; western blot, 1/1000; immunocytochemistry, 1/200), rabbit anti-phospho VEGFR2 (Tyr^1175^) (Cell Signaling, 2478; RRID, AB_331377; western blot, 1/1000), rabbit anti-phospho VEGFR2 (Tyr^951^) (Cell Signaling, 2471; RRID, AB_331021; western blot, 1/1000), goat anti-VEGFR2 (R&D, AF644; RRID, AB_355500; western blot, 1/1000), rabbit anti-phospho c-Src (Tyr^416^) (Cell Signaling, 2101; RRID, AB_331697; western blot, 1/1000), rabbit anti-phospho c-Src (Tyr^418^) (Invitrogen, 44660G; RRID, AB_2533714; immunocytochemistry, 1/100), rabbit anti-Erg1/2/3 (Abcam, ab92513; RRID, AB_2630401; immunohistochemistry, 1/200), mouse anti-VE-cadherin-Alexa647 (BD Biosciences, 561567, RRID:AB_10712766, immunocytochemistry, 1/250), rabbit anti-phospho-paxillin (Tyr^118^) (Invitrogen, 44-722G; RRID, AB_2533733; western blot, 1/1000, immunocytochemistry, 1:200), rabbit anti-phospho FAK (Tyr^576^) (Cell Signaling, 3281; RRID, AB_331079; western blot, 1/1000), mouse anti-FAK (Santa Cruz, sc-271126; RRID, AB_10614323; western blot, 1/1000), rabbit anti-phospho c-Src (Tyr^527^) (Cell Signaling, 2105; RRID, AB_331034; western blot, 1/1000), rabbit anti-GAPDH (Cell Signaling, 2118; RRID, AB_561053; western blot, 1/5000), mouse anti-fibronectin (BD Biosciences, 610077; RRID, AB_2105706; immunocytochemistry, 1/200), rat anti-F4/80 (Bio-Rad, MCA497R; RRID, AB_323279; immunohistochemistry, 1/100), rat anti-Ter119 (Invitrogen, 14-5921-81; RRID, AB_467726; immunohistochemistry, 1/200), rabbit anti-NG2 (Millipore, AB5320, RRID:AB_11213678, immunohistochemistry, 1/200) rabbit anti-Collagen IV (Abcam, AB6586, AB_305584, immunohistochemistry, 1/500), rabbit anti-Ki-67 (Abcam, 15580; RRID, AB_443209; immunohistochemistry, 1/200), rabbit anti-cleaved caspase 3 (Cell Signaling, 9661; RRID, AB_2341188; immunohistochemistry, 1/200), mouse anti-Yes (BD Biosciences, 610376; RRID, AB_397759; WB, 1/1000) and mouse anti-FYN (BD Biosciences, 610163; RRID, AB_397564; WB, 1/1000).

Fluorescently labeled secondary antibodies were obtained from Invitrogen (immunohistochemistry, 1/250; immunocytochemistry, 1/400). Horseradish peroxidase (HRP)-labeled secondary antibodies were obtained from ThermoFisher Scientific (western blot, 1/5000).

### Mice

Animals were propagated at the local animal facility under laminar airflow conditions with a 12 h light/dark cycle at a temperature of 22-25°C. All animal work was approved by the Uppsala University board of animal experimentation (permit 5.2.18-8927-16) and The University of Queensland's Molecular Biosciences Animal Ethics Committee (permits IMB424/17 and IMB231/17/BREED). *mTmG* mice were obtained from The Jackson Laboratory (Muzumdar et al., 2007). *TdTomato* mice were obtained from Prof. Benjamin Hogan (Peter MacCallum Cancer Centre, Australia) and The Jackson Laboratory (Madisen et al., 2010). *Cdh5-CreERT2* mice were provided by Ralf Adams (MPI, Münster, Germany) (Kogata et al., 2006; Wang et al., 2010). Lifeact-EGFP mice were provided by Roland Wedlich-Söldner (University of Münster, Germany) (Riedl et al., 2010). *c-Src^−/−^* global knockout mice were obtained from The Jackson Laboratory (Soriano et al., 1991). c-Src-floxed mice were delivered from the Nice Mice, National Resource Center for Mutant Mice, Model Animal Research Center, China.

### Inducible gene deletion

Cre activity and gene deletion were induced by intraperitoneal injections to pups (male and female) with 100 μg tamoxifen (Sigma, T5648) at P1, P2 and P3, and mice were sacrificed at P5 or P23. Tamoxifen (30 μg) was injected at P1 to induce mosaic deletion and mice were sacrificed at P5.

### Immunohistochemistry (IHC)

Eyes were removed and prefixed in 4% paraformaldehyde (PFA) for 20 min at room temperature. Tracheas were fixed in 4% PFA for 15 min at room temperature. After dissection, tissues were blocked overnight at 4°C in 1% FBS (Gibco), 3% BSA (Sigma) and 0.5% Triton X-100 (Sigma). Samples were incubated overnight with primary antibodies in blocking reagent, followed by washing and incubation with the appropriate secondary antibody for 2 h at room temperature, and mounted in fluorescent mounting medium (ProLong Gold Antifade, ThermoFisher). Endothelial cells were treated with 80 ng/ml mouse VEGFA164 for 10 min before permeabilization with 3% PFA, 0.5% Triton X100 in PBS for 3 min, followed by fixation in 3% PFA in PBS for 15 min. Antibodies were added in 5% BSA and 5% donkey serum in PBS. Images were acquired using a Zeiss LSM700 confocal microscope. For comparison purposes, different sample images of the same antigen were acquired under constant acquisition settings.

For staining with phospho-antibodies, animals were anaesthetized with ketamine:xylazine mixture (1:4). Using a peristaltic pump, cardiac perfusion was performed with 2 ml of PBS then 2 ml of fixative [1% PFA, 0.1% triethanolamine, 0.1% Triton X100, 0.1% NP-40 (pH 7.5)]. Eyes were removed and fixed for another 2 h at room temperature. After dissection, retinas were blocked for 2 h with shaking at room temperature [0.5% Triton-X100, 0.05% sodium deoxycholate, 1% BSA, 2% FBS and 0.02% sodium azide prepared in PBS (pH=7.4)]. Samples were incubated overnight with primary antibodies in blocking reagent, diluted in one part block:one part 1×TBS, followed by washing for 3×1 h at room temperature with TBS 0.2% Triton. Secondary antibodies were added overnight at 4°C with slow agitation in one part buffer B:one part 1×TBS, followed by washing for 3×1 h at room temperature with TBS 0.2% Triton and were mounted using fluorescent mounting medium (Fluoromount, ThermoFisher).

### Hyaloid vasculature analysis

Hyaloid vessels were dissected from P5 eyes. Eyeballs were removed and fixed for 20 min in 4% PFA at room temperature. Using an insulin syringe, eyeballs were injected with 5% gelatin (Sigma) in PBS around the cornea into the eyeball space, four injections of ∼50-100 µl total. Eyeballs were left on ice for 30-45 min to allow the gelatin to set, before removal of the remaining eye tissue to isolate the gelatin plug (containing the hyaloid plexus). The hyaloid plexus plug was transferred to a glass slide, before addition of antibodies for 20 min at room temperature, rinsed with PBS and then mounted in fluorescent mounting medium (ProLong Gold Antifade with DAPI, ThermoFisher).

### Immunocytochemistry

Immunofluorescent staining was in general performed on HUVECs cultured on 12 mm glass coverslips coated with 5 µg/ml FN (Sigma) or 10 µg/ml vitronectin (Sigma) until confluent, washed with PBS^+/+^(supplemented with 1 mM CaCl_2_ and 0.5 mM MgCl_2_), fixed in 4% PFA (Sigma), and blocked for 30 min with 3% BSA and 0.3% TritonX-100 (Sigma). Primary antibodies were incubated in 1.5% BSA for 60 min at room temperature, followed by washing and incubation with the appropriate secondary antibody for 45 min at room temperature and mounting in ProlongGold+Dapi solution (Cell Signaling Technologies). *Z*-stack image acquisition was performed on a confocal laser scanning microscope (Zeiss LSM880) using a 40× NA 1.3 or 63× NA 1.4 oil immersion objective.

### VE-cadherin patching image analysis

VE-cadherin junctional patterning was assessed using a blinded image analysis approach. 3D retinal image stacks were processed for VE-cadherin morphology, into ‘patches’ of 16×16 µm. Images were acquired with a Zeiss LSM700 confocal microscope using ×63 numerical aperture 1.4 objectives. The VE-cadherin morphology in each patch was hand-classified on a scale of 1 [‘active’ (irregular/serrated morphology with diffuse/vesicular regions, color labeled red in output images)] to 6 [inactive’ (straighter morphology with less vesicular staining, color labeled blue in heatmap images)]. Heatmap images were generated using specialized Matlab software described previously (Bentley et al., 2014).

### Image analysis

Phospho-VE-cadherin analysis was performed using ImageJ. First, total VE-cadherin was thresholded and the area measured and saved as a mask. Phospho-VE-cadherin was then thresholded, masked with the total VE-cadherin and total area was measured.

Focal adhesion analysis (Fig. 7) was performed using ImageJ. p-Paxillin staining was thresholded and VE-cadherin was used to draw two or three cells per image and added as ROI. Subsequent particle analysis of each ROI was used to measure particle number, size and density per cell.

Phospho(p)-Paxillin retinal analysis was performed using Imaris (Bitplane). Vascular area was defined using Isolectin-B4 staining to define a 3D mask. Within the vascular mask, the Sum Intensity of p-Paxillin was recorded and normalized to the total area of the vascular mask. To account for variations in staining intensity between samples, two non-vascular areas per image were analyzed for p-Paxillin, and the SumInt/area of the vascular mask was normalized to the average of the non-vascular SumInt/area. Three or four images were quantified per retina and averaged to determine the p-Paxillin intensity per retina (n is one retina).

Quantification of endothelial cell proliferation, Collagen IV empty sleeves, VE-cadherin phosphorylation and phospho-Paxillin were all performed at the plexus region at the vascular front of the retina. When imaging, arterial and venous regions were carefully avoided. All images shown are representative of images that were quantified.

### Isolation of lung endothelial cells

Endothelial cells were isolated as described previously (Li et al., 2016). Mouse lungs were collected at P10, and minced and digested in 10 ml Dulbecco's PBS medium containing 2 mg/ml 1 collagenase type I (Sigma), for 1 h at 37°C with shaking, followed by filtration through a 70 µm disposable cell strainer (BD Falcon). Cells were centrifuged at 400 ***g*** for 8 min at 4°C, suspended in ice-cold PBS with 0.1% bovine serum albumin (BSA) and incubated with anti-rat immunoglobulin G-coated magnetic beads (Dynabeads sheep anti-Rat IgG, Invitrogen) pre-coupled with rat anti-mouse CD31 for 10 min, with gentle agitation. Beads were separated using a magnetic particle concentrator (Dynal MPC-S, Invitrogen). The beads were washed with PBS and endothelial cells were suspended in EGM-2 plus medium, supplemented with singlequots (Lonza CC-5035). To induce Cre activity, cells were treated with 1 µM of 4-hydroxytamoxifen (Sigma).

### Metatarsal assay

Metatarsals were isolated from E16.5 mice using a protocol adapted from Song et al. (2015). After dissection, one metatarsal per well was placed in a µ-Plate 24 well ibiTreat plate with a 1.5 polymer coverslip (Ibidi) and left in 170 µl of MEM-alpha (Gibco) with 10% FCS and 1% penicillin/streptomycin (Sigma). After 3 days, media were replaced with 300 µl MEM-alpha + 10% FCS + 1% pen/strep per well and media changed every 48 hours. To induce Cre activity, cells were treated with 1 µM of 4-hydroxytamoxifen (Sigma) after 5 days. After 14 days, metatarsals were imaged every minute over 4 h using a Zeiss LSM700 confocal microscope with ×63 numerical aperture 1.4 objectives. Alternatively, metatarsals were fixed in 4% PFA in PBS for 20 min and antibodies were added in 3% Trixon X-100, 1% Tween and 0.5% BSA in PBS.

### Cell culture and treatments

Human umbilical vein endothelial cells (HUVECs) purchased from Lonza (CC-2935) were cultured until passage 5 in EGM-2 plus medium, supplemented with singlequots (Lonza CC-5035). Overnight starvation with EBM-2 plus basal medium, followed by stimulation with recombinant human VEGFA (100 ng/ml; R&D Systems) for the indicated duration. Human embryonic kidney (HEK)-293T cells were maintained in DMEM with L-glutamine and sodium pyruvate (Invitrogen), containing 10% (v/v) heat-inactivated fetal bovine serum (GE Healthcare Australia), 100 U/ml penicillin and streptomycin (Life Technologies Australia). All cells were cultured at 37°C in 5% CO_2_.

c-Src-GFP was kindly provided by Dr Margaret Frame (University of Edinburgh, UK). The c-Src fragment was obtained by digesting the c-Src-GFP plasmid with XhoI and BamHI, and inserting into pmKate2-N plasmid (Evrogen). The c-Src-mKate2 was then subcloned into the pLenti-MP2 plasmid (Addgene plasmid 36097) and mKate2 alone was subcloned into pLenti-MP2 as control for lentiviral construction. Lentiviral constructs were packaged into lentivirus in HEK-293T cells by means of third generation lentiviral packaging plasmids (Dull et al., 1998). Lentivirus containing supernatant was harvested on days 2 and 3 after transfection. Lentivirus was concentrated using a Lenti-X concentrator (Clontech, 631232). HEK cells were transfected with the expression vectors according to the manufacturer's protocol with PEI 2500 (BioScientific) and lentiviral-transduced target HUVECs were used for assays after 48-72 h.

siRNA targeting c-Src (VPDSIRNA2D, SASI_Hs01_00112907) and scrambled non-silencing control siRNA (SIC001) both used at working concentration of 20 nM were purchased from Sigma. siRNA transfections were performed according to manufacturer's protocol using Polyplus INTERFERin (In Vitro Technologies) and cells were used for assays after 48 h.

### Western blotting

After washing once with PBS+/+ (1 mM CaCl and 0.5 mM MgCl), cells were lysed in 95°C SDS-sample buffer containing 4% β-mecapto-ethanol. Samples were boiled at 95°C for 5-10 min to denature proteins. Proteins were separated on 4-15% mini protean TGX precast gel (Bio-Rad) in running buffer [200 mM glycine, 25 mM Tris and 0.1% SDS (pH 8.6)], transferred to nitrocellulose membrane (Bio-Rad, 1620112) in blot buffer (48 nM Tris, 39 nM glycine, 0.04% SDS and 20% methanol) and subsequently blocked with 5% (w/v) BSA (Sigma) in Tris-buffered saline with Tween 20 (TBST) for 30 min. The immunoblots were analyzed using primary antibodies incubated overnight at 4°C and secondary antibodies linked to horseradish peroxidase (HRP) (ThermoFisher Scientific) incubated for 1 h at room temperature, after each step immunoblots were washed four times with TBST. HRP signals were visualized by enhanced chemiluminescence (ECL) (Bio-Rad) and imaged with Chemidoc.

## Supplementary Material

Supplementary information

Reviewer comments
