# Supplementary material for: c-Src controls stability of sprouting blood vessels in the developing retina independently of cell-cell adhesion through focal adhesion assembly
Source: Development. 2020 Apr 6;147(7):dev185405. doi: 10.1242/dev.185405 (PMC7157583; doi:10.1242/dev.185405)
Supplement: Supplementary information [file develop-147-185405-s1.pdf]

Supplemental Information

Figure S1

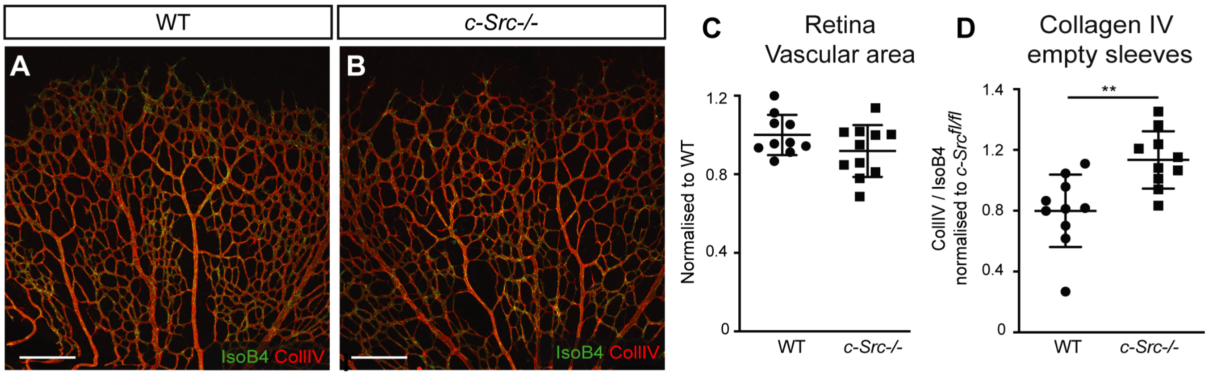

Figure S1. Analysis of global c-Src knockout mice.

(A, B) WT and *c-Src-/-* mice were analysed for retinal defects at P6. Scale bar, 200 μm.

(C) Moderate but not significant defects were observed in total vascular area.

(D) *c-Src-/-* retinas at P6 display increased number of empty collagen IV sleeves in the retinal vasculature compared to WT littermates. n > 10, n is the number of retinas.

** $p < 0.01$. Error bars represent mean ± SEM. Statistical significance was determined using a Mann-Whitney test

Development • Supplementary information

Figure S2

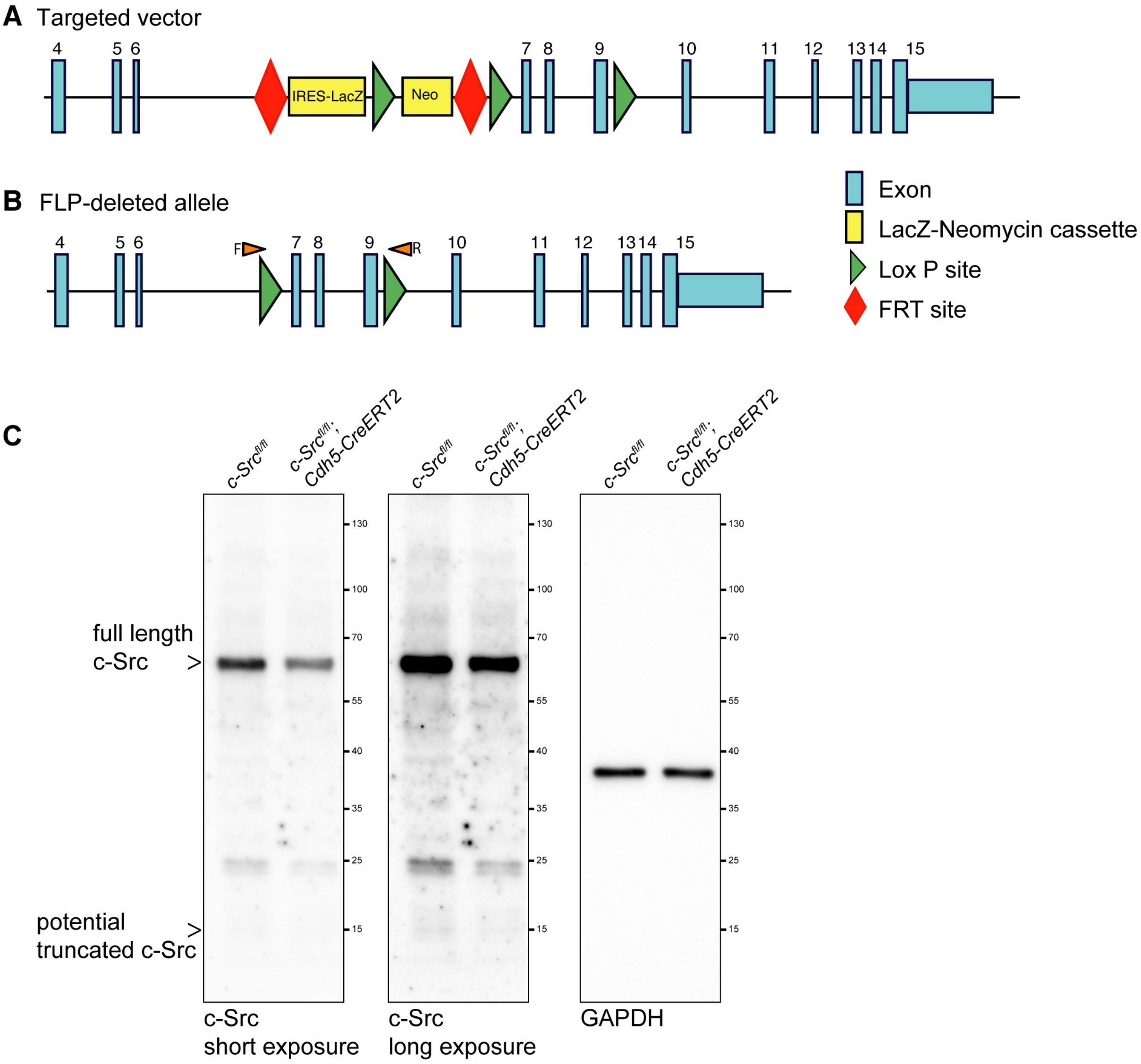

Figure S2. Generation of c-Src floxed mice.

(A) *c-Src-floxed* mice were generated through homologous recombination and insertion of a FRT-SA-IRES-LacZ-loxP cassette targeted to the 5' upstream of exon 7 in the Src (AW259666; pp60c-src) gene (gene ID:20779). A single loxP site was inserted downstream of exon 9.

(B) To render the mouse conditional, the KO mice were crossed with FLP deleted mice, causing global excision of the FRT sites, with two loxP sites remaining. The deletion results in deletion of exon 7,8,9 and a frame shift mutation in exon 10.

(C) Western blot assessment of c-Src protein expression in endothelial cells isolated from lungs of *c-Src*$^{flox/flox}$ and *c-Src*$^{flox/flox}$*; Cdh5-CreERT2* mice showed no presence of predicted truncated 15.7 kDa c-Src protein after induction of c-Src deletion.

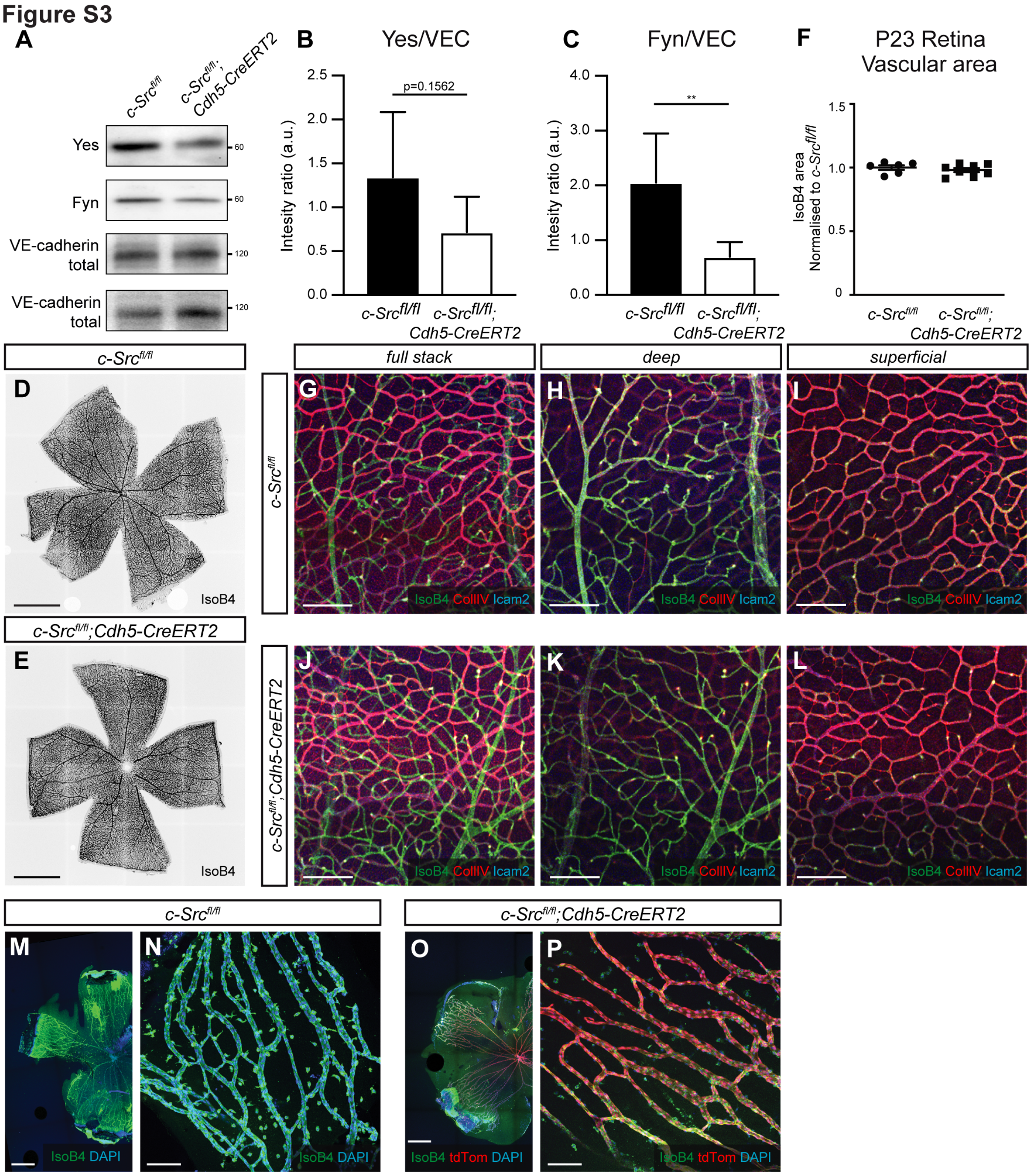

Figure S3. Analysis of *c-Src$^{flox/flox}$; Cdh5-CreERT2* mice.

(A) Endothelial cells were isolated from lungs of *c-Src$^{flox/flox}$* and *c-Src$^{flox/flox}$; Cdh5-CreERT2* mice and protein expression was assessed by western blot.

(B, C) While no significant change was detected in Yes expression, Fyn expression decreased in c-Src deficient mice. n>5 mice.

(D, E) Vasculature of retinas from P23 mice immunostained with Isolectin B4. Scale bar, 1000 µm.

(F) *c-Src*-deficient retinas display no reduced vascular area. n>6, n is the number of retinas.

(G-L) Vasculature of *c-Src$^{flox/flox}$* and *c-Src$^{flox/flox}$; Cdh5-CreERT2* retinas from P23 mice

Development • Supplementary information

immunostained with Isolectin B4, Collagen IV and Icam2 at different depths showed no difference in the fully remodelled retinal vasculature. Scale bar, 100 μm.

(M-P) Hyaloid vasculature of *c-Src*$^{flox/flox}$ and *c-Src*$^{flox/flox}$*; tdTomato; Cdh5-CreERT2* mice at P5. Scale bar, 500 μm in M and O, 100 μm in N and P. Images are representative of n>6 mice. ** $p < 0.01$. Error bars represent mean ± SEM. Statistical significance was determined using a Mann-Whitney test.

Development • Supplementary information

Figure S4

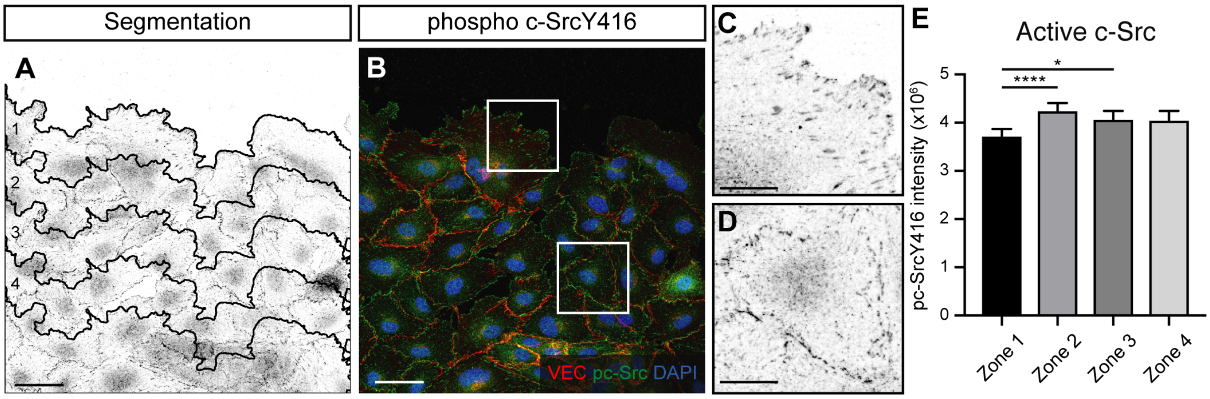

Figure S4. phospho-c-Src Y416 in migrating HUVECs.

(A) HUVECs migrating for 3 hours after scratching were segmented into 4 zones of 50 μm starting at the migration front.

(B) HUVECs were immunostained with phospho-c-Src Y416 to show active c-Src, VE-cadherin and DAPI. Scale bar, 50 μm.

(C, D) Higher magnification images of boxed areas in B show subcellular distribution of phospho-c-Src Y416 at the vascular front (C) and at junctions (D). Scale bar, 20 μm.

(E) c-Src activity based on phospho-c-Src Y416 intensity in the 4 zones in A showed a significant increase in staining intensity in the second and third zone compared to the migration front in zone 1. n=21 images from 2 independent experiments.

* $p < 0.0180$, **** $p < 0.0001$. Error bars represent mean ± SEM. Statistical significance was determined using a one-way ANOVA with Holm-Sidak's multiple comparisons test.

Development • Supplementary information

Figure S5

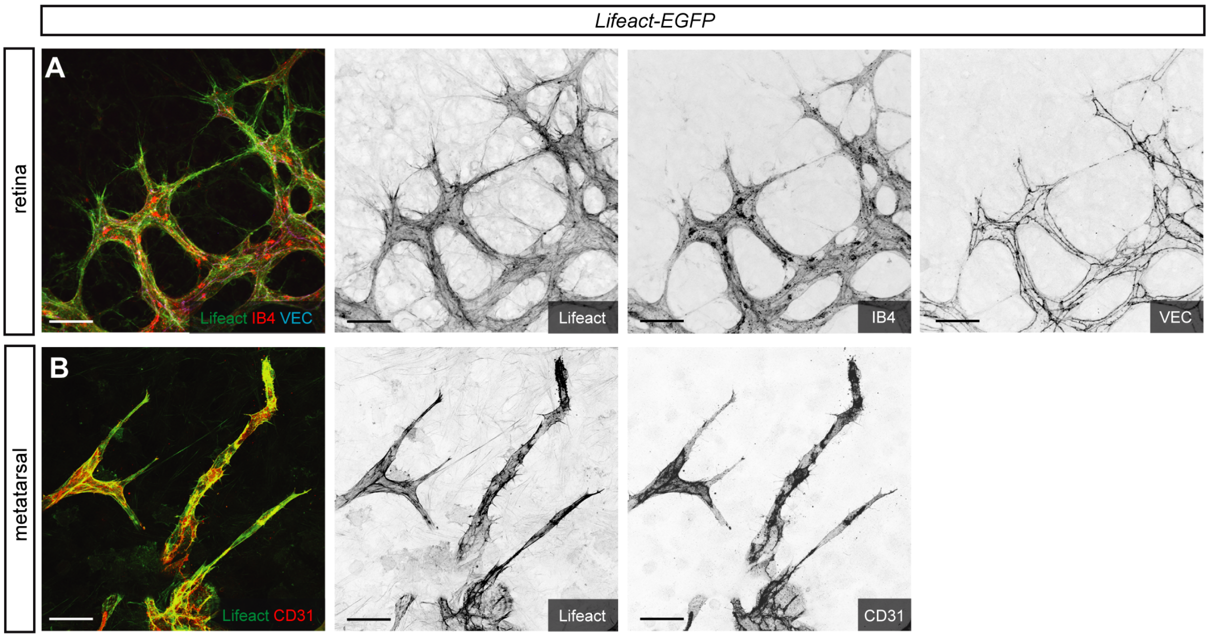

Figure S5. Lifeact-EGFP is highly expressed in endothelial cells.

(A) Lifeact-EGFP vessels in the retina co-stained with Isolectin B4 and VE-cadherin, confirming their endothelial identity. Scale bar, 25 µm.

(B) Lifeact-EGFP vessels in metatarsals co-stained with CD31, confirming their endothelial identity. Scale bar, 25 µm.

Figure S6

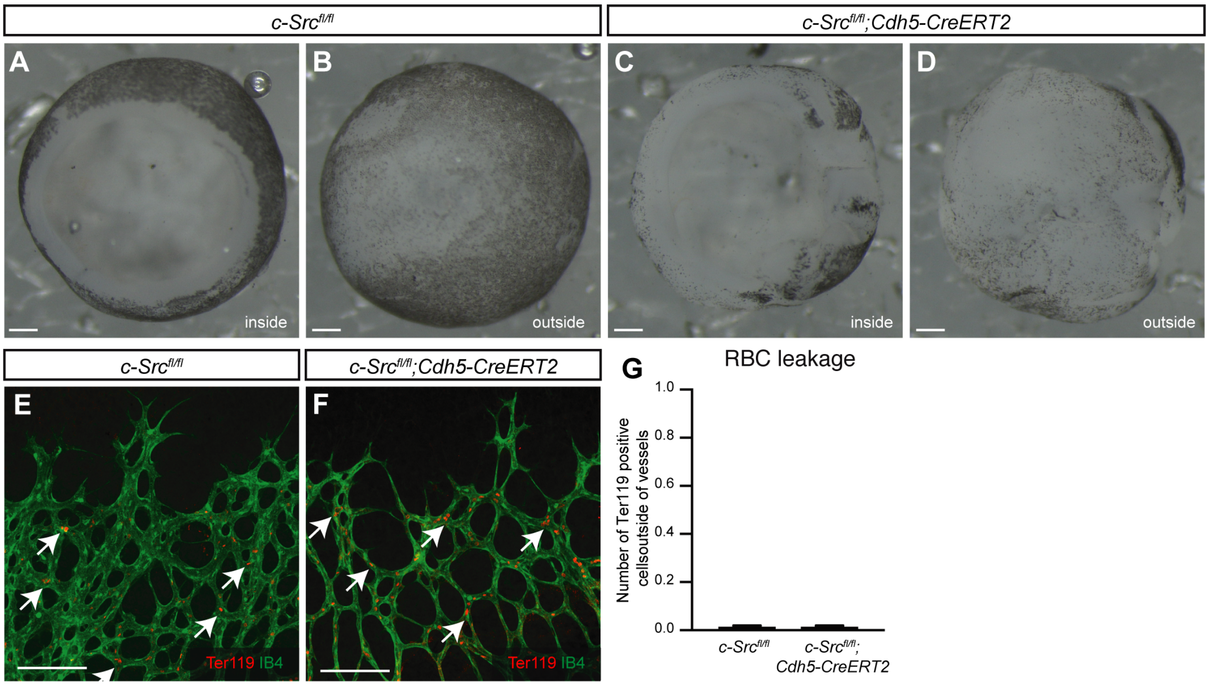

Figure S6. No haemorrhage is observed in c-Src-deficient retinas.

(A-D) *c-Src*$^{flox/flox}$ and *c-Src*$^{flox/flox}$*; Cdh5-CreERT2* retinas after dissection showed no bleeding at P6. Scale bar, 200 μm.

(E, F) *c-Src*$^{flox/flox}$ and *c-Src*$^{flox/flox}$*; Cdh5-CreERT2* retinas immunostained for Ter119 (red) to visualise red blood cells (indicated with white arrows) and Isolectin B4 (green) at P6. Scale bar, 100 μm.

(G) No red blood cells were detected outside of the vasculature in *c-Src*$^{flox/flox}$ or *c-Src*$^{flox/flox}$*; Cdh5-CreERT2* retinas. n>4, n is the number of retinas.

Error bars represent mean ± SEM. Statistical significance was determined using a Mann-Whitney test.

Development • Supplementary information

Figure S7

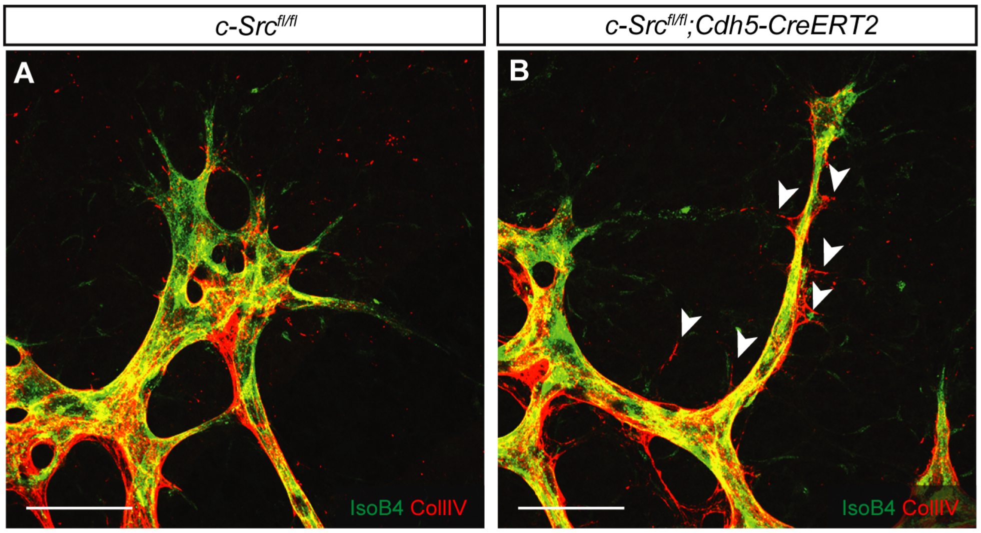

Figure S7. Empty collagen IV sleeves at sprouting front.

(A, B) Representative images of empty Collagen IV sleeves in *c-Src*$^{flox/flox}$ and *c-Src*$^{flox/flox}$*; Cdh5-CreERT2* retinas at the sprouting front. CollIV empty sleeves are abundant at the vascular front. Arrowheads indicate empty Collagen IV sleeves. Scale bar, 50 µm.

Development • Supplementary information

Figure S8

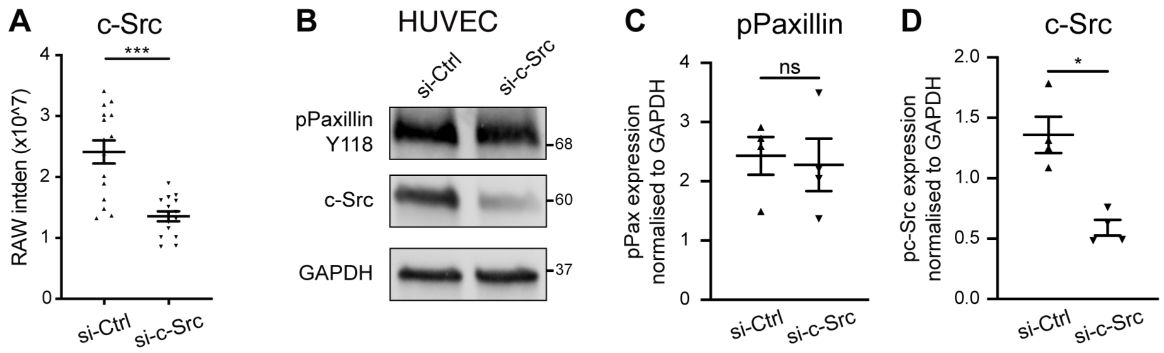

Figure S8. c-Src protein knockdown after siRNA.

(A) A significant decrease in c-Src expression upon transfection with siRNA based on c-Src staining intensity. n>30 cells in total, from 3 independent experiments. Intensity measurements from cells used in Figure 7 C-F.

(B-D) Western blotting of HUVECs used in Figure 7L-P showed no significant decrease in p-PaxillinY118, while c-Src protein expression was significantly reduced upon siRNA transfection. n=4 from 4 independent experiments.

* $p < 0.05$, *** $p < 0.001$. Error bars represent mean ± SEM. Statistical significance was determined using a Mann-Whitney test.

Development • Supplementary information

Figure S9

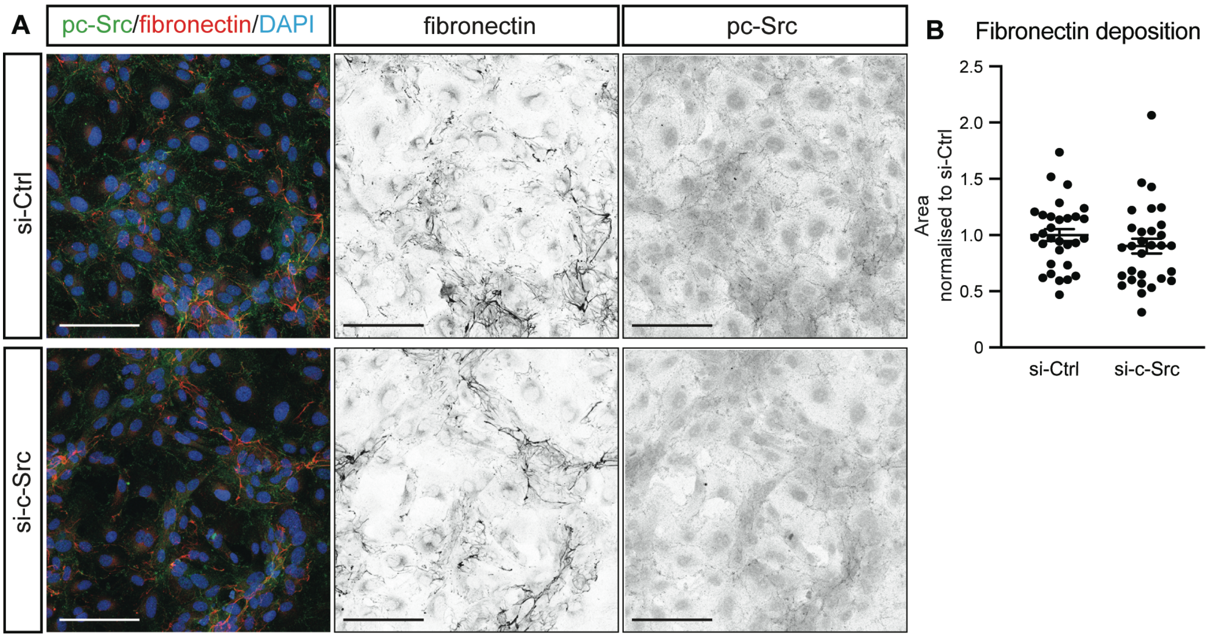

Figure S9. Fibronectin deposit by Src depleted HUVECs.

(A) HUVECs transfected with control or c-Src siRNA were cultured on non-coated glass and stained with phospho-c-Src Y416, fibronectin and DAPI. Scale bar, 100 μm.

(B) Quantification of fibronectin deposit area showed no significant difference between control and c-Src depleted cells. Area was determined by thresholding fibronectin staining and measuring using ImageJ. n= 30 images from 3 independent experiments.

** $p < 0.01$. Error bars represent mean ± SEM. Statistical significance was determined using a Mann Whitney test.
