# Supplementary material for: c-Src controls stability of sprouting blood vessels in the developing retina independently of cell-cell adhesion through focal adhesion assembly
Source: Development. 2020 Apr 6;147(7):dev185405. doi: 10.1242/dev.185405 (PMC7157583; doi:10.1242/dev.185405)
Supplement: Reviewer comments [file develop-147-185405_review_history.pdf]

c-Src controls stability of sprouting blood vessels in the developing retina independently of cell-cell adhesion through focal adhesion assembly.

Lilian Schimmel, Daisuke Fukuhara, Mark Richards, Yi Jin, Patricia Essebier, Emmanuelle Frampton, Marie Hedlund, Elisabetta Dejana, Lena Claesson-Welsh and Emma Gordon
DOI: 10.1242/dev.185405

Editor: Patrick Tam

Review timeline

Original submission:	9 October 2019
Editorial decision:	12 November 2019
First revision received:	4 February 2020
Accepted:	19 February 2020

Original submission

First decision letter

MS ID#: DEVELOP/2019/185405

MS TITLE: c-Src controls stability of sprouting blood vessels in the developing retina independently of cell-cell adhesion through focal adhesion assembly.

AUTHORS: Lilian Schimmel, Daisuke Fukuhara, Mark Richards, Yi Jin, Elisabetta Dejana, Lena Claesson-Welsh, and Emma Gordon

I have now received two referees' reports on the above manuscript. The report of the third referee is pending, which will be forwarded to you when it is available. The referees' comments are appended below, or you can access them online: please go to BenchPressand click on the 'Manuscripts with Decisions' queue in the Author Area. As it has been more than 4 weeks since the submission of this manuscript, I have moved to make a decision on this submission based on the two reports that were received to this date.

As you will see, the referees express considerable interest in your work, but have some significant criticisms and recommend a substantial revision of your manuscript before we can consider publication. If you are able to revise the manuscript along the lines suggested, which may involve further experiments, I will be happy receive a revised version of the manuscript. Your revised paper will be re-reviewed by one or more of the original referees, and acceptance of your manuscript will depend on your addressing satisfactorily the reviewers' major concerns. Please also note that Development will normally permit only one round of major revision.

Please attend to all of the reviewers' comments (and the critiques of the third reviewer if they are available before the submission of the revised manuscript), and ensure that you clearly highlight all changes made in the revised manuscript. Please avoid using 'Tracked changes' in Word files as these are lost in PDF conversion. I should be grateful if you would also provide a point-by-point response detailing how you have dealt with the points raised by the reviewers in the 'Response to Reviewers' box. If you do not agree with any of their criticisms or suggestions please explain clearly why this is so.

© 2020. Published by The Company of Biologists under the terms of the Creative Commons Attribution License (https://creativecommons.org/licenses/by/4.0/).

Reviewer 1

Advance summary and potential significance to field

This study used both in vivo and ex vivo models of angiogenesis to assess the effects of c-Src on sprout formation and vessel stabilization during vascular development. The data showed that endothelial-specific deletion of c-Src is associated with discrete changes at the front of sprouting vessels. the Vascular alterations include reduced filopodial extensions of tip cells, reduced junctional density, destabilization of vessel sprouts and directional sprout movements, increased vessel regression and impaired focal adhesion at cell-matrix junctional points. The data suggest that the inability of c-Src-deficient endothelial cells to form a stable cell-matrix connections causes the reduced vascular density and increased vascular regression.

Comments for the author

-The study appears to be limited in scope as it focuses on the overall vascular phenotype in c-Src-deficient endothelial cells during the formation of superficial capillary plexus in the retina only. Additional experiments are needed to assess the long term effects of c-Src deletion on the fully remodeled retinal vasculature in adult mice. This can be done by inducing c-Src deletion at P1 and assessing the vascular phenotype in the adult retina wherein vascular remodeling has been completed (e.g., P24, P28).
- Loss of c-Src function may have caused important changes at the cell-cell interaction points as well since it was associated with reduced Fyn and YAP levels. Consequently, cell-cell junctions may be disrupted as well. It is important to determine whether vascular permeability at the destabilized vascular front is impaired or not. This should be done through systemic injection of labeled tracers.
- While a major focus is placed on proteins involved in cell-cell junctions (i.e., paracellular transport), it is important to also assess whether the transcellular transport is affected or not by loss of c-Src function in endothelial cells.
-Since the data suggest that endothelial loss of c-Src causes increased vascular regression, it is important to confirm this observation by assessing whether regression of the hyaloid vessels is affected or not upon loss of c-Src gene function.
- Since previous studies have shown that mice with global deletion of c-Src develop osteopetrosis due to impaired osteoclast function, it is important to assess the indirect effects of c-Src deletion on macrophages. The latter play an important role in vessel regression/remodeling during development.
-Does loss of c-Src function affect pericyte recruitment to developing blood vessels as well?
- Angiogenic sprouting process and mechanisms in the retina is different form that in tracheal and metatarsal explants as the cellular components involved are different. Therefore, caution must be taken when extrapolating data obtained in these two different biological systems.
- The role of integrins is discussed in the discussion section of the paper but no data has been provided concerning the role of integrins in cell-matrix interactions in c-Src-deficient endothelial cells especially since cell-matrix interactions are affected by loss of c-Src function.
- Since c-Src is an intracellular protein and since its global deletion increases vascular permeability according to the literature, it is unclear why its endothelial-specific deletion had no effects on paracellular permeability of blood vessels.

Reviewer 2

Advance summary and potential significance to field

The manuscript by Schimmel and colleagues addresses the regulation of angiogenesis in the postnatal retina by the kinase c-Src. The authors propose that c-Src controls vessel growth through cell-matrix interactions and not through changes in vascular integrity.

The main findings of this study are: (1) c-Src is required for sprouting angiogenesis in retina by regulating focal adhesion assembly and cell-matrix adhesion, (2) c-Src has no overt effect on endothelial cell-cell adhesion, and (3) c-Src signaling acts differently on adherens junctions and focal adhesions.

© 2020. Published by The Company of Biologists under the terms of the Creative Commons Attribution License (https://creativecommons.org/licenses/by/4.0/).

These results improve our understanding of the mechanisms controlling vascular growth and, given the important roles of Src singling in many different processes, findings should be of interest to many researchers.

Comments for the author

Overall, data quality and presentation are good, but insight into the underlying mechanism is limited. It is also noted that the manuscript could be improved by editing (e.g. see first sentence of the abstract) and the Discussion should be shortened by taking out aspects that are of peripheral relevance.

Specific criticism:

1. Conditional deletion of c-Src in endothelial cells results in deletion of exons 7-9 and a frame shift mutation in exon 10. It would be important that authors check whether a truncated protein is formed after c-Src gene deletion, which may act as a dominant negative, kinase-deficient form. The antibody used in this study to detect c-Src was raised against epitopes 82-169 of chicken v-Src and should therefore recognize a potential truncated variant comprised of SH3 and SH2 domains.

2. While the role of c-Src in sprouting angiogenesis is well addressed, phospho-Src immunostaining is not shown in any experiment in vitro or in vivo. This information is crucial for understanding the role of Src in angiogenesis. How is active Src distributed between tip and stalk cells? Is it polarized and concentrated at the leading edge of tip cells? Is there is localized heterogeneity (as shown in vitro by Vera RH et al, Tissue Engineering, 2009) or differential expression in vessel subtypes?

3. The authors should provide detailed descriptions on how quantitation was done. Without this information, one cannot properly assess whether EC proliferation was unaffected in the c-Src mutant retinal plexus. As the role of Src in the regulation of cell proliferation was already shown, the authors should provide a deeper analysis of proliferation in different vascular beds, such as the capillary vascular plexus and the vein region. As mentioned above, it would be helpful to correlate these results with the localization of phosphorylated c-Src.

4. The authors found that c-Src has no effect on tip cell identity and does not regulate sprouting. Furthermore, deletion of c-Src resulted in reduced vessel stability in the angiogenic front area but also in the vascular plexus below (as indicated in Fig. 3J, K). The authors should provide the information about the regions where quantitations were done. In light of the vascular phenotype, it is not overly surprising that VE-cadherin in junctions of the angiogenic front area was not altered. Instead, the authors might want to carefully analyze VE-cadherin positive adherens junctions and VE-cadherin phosphorylation in the plexus region with increased collagen IV + empty sleeves.

5. Murine lung ECs were immunoselected using CD31 beads and these cells are likely to represent a mixture of different EC populations. To rule out completely that c-Src plays no role in VE-cadherin phosphorylation (which might have been overlooked by diluting the effect on c-Src using mixed EC populations) and internalization, the authors could investigate VE-cadherin phosphorylation in a wound assay using primary cells such as HUVECs.
The pVEC 658 blot could be added to Fig. 4H.

6. In Figure 6 A and B pc-SrcY416/ c-Src total were increased in si-Src knockdown cells, maybe as compensatory effect, as suggested by the authors. This antibody is not entirely specific for pSrc 416 and detects other activated tyrosine kinases (as is also stated by the manufacturer). Therefore, the authors should validate antibody specificity by confirming that levels of pSrc are reduced when cells are treated with a Src family inhibitor, such as PP2.

7. The fact that c-Src regulates paxillin phosphorylation in vivo and the formation of focal adhesions is interesting. The main message of this study is that c-Src regulates sprouting by regulating focal adhesion assembly and cell-matrix adhesion. The authors nicely discuss potential mechanisms, which require further investigation. It was previously shown that not only focal adhesion formation but also fibronectin fibrillogenesis and actin organization depend on the phosphorylation state of paxillin (Zeidel-Bar R. et al., JCS, 2007). Culture of cells on glass without coating by matrix molecules might be therefore problematic for same of the assays.

© 2020. Published by The Company of Biologists under the terms of the Creative Commons Attribution License (https://creativecommons.org/licenses/by/4.0/).

8. In Fig. 6, it is surprising and, based on the statements in the introduction, a bit counter-intuitive that VEGF stimulation does not lead to the activation of Src or FAK relative to the 0min time point. Please explain.

First revision

Author response to reviewers' comments

Reviewer 1 Advance Summary and Potential Significance to Field:
This study used both in vivo and ex vivo models of angiogenesis to assess the effects of c- Src on sprout formation and vessel stabilization during vascular development. The data showed that endothelial-specific deletion of c-Src is associated with discrete changes at the front of sprouting vessels. the Vascular alterations include reduced filopodial extensions of tip cells, reduced junctional density, destabilization of vessel sprouts and directional sprout movements, increased vessel regression and impaired focal adhesion at cell-matrix junctional points. The data suggest that the inability of c-Src-deficient endothelial cells to form a stable cell-matrix connections causes the reduced vascular density and increased vascular regression.

We thank the reviewer for this comprehensive summary of our findings.

R1.1. *The study appears to be limited in scope as it focuses on the overall vascular phenotype in c-Src-deficient endothelial cells during the formation of superficial capillary plexus in the retina only. Additional experiments are needed to assess the long term effects of c-Src deletion on the fully remodelled retinal vasculature in adult mice. This can be done by inducing c-Src deletion at P1 and assessing the vascular phenotype in the adult retina wherein vascular remodelling has been completed (e.g., P24, P28).*

Response: We agree these experiments are a valuable contribution to the manuscript and they have been included in Supplementary Figure 3D-L. We induced deletion of c-Src by injection with tamoxifen at P1,2 and 3, then left mice until P23 for retinal analysis. Our results show no changes in the retinal vasculature within the superficial or deep plexus in c-Src deficient mice at this late stage of development, suggesting that the vascular defects observed at P5 are transient, and loss of c-Src is able to be compensated for with time. This has been commented on within the text on page 6.

R1.2. *Loss of c-Src function may have caused important changes at the cell-cell interaction points as well since it was associated with reduced Fyn and YAP levels. Consequently, cell- cell junctions may be disrupted as well. It is important to determine whether vascular permeability at the destabilized vascular front is impaired or not. This should be done through systemic injection of labeled tracers.*

Response: To try and address this question and to show that loss of c-Src does not result in a complete breakdown of cell-cell junctions and haemorrhage from vessels, we have imaged freshly dissected retinas to reveal no leakage of blood from vessels (Supplementary Figure S6A-D). We have also performed immunostaining with red blood cell marker Ter119 and not observed any RBC leakage in c-Src-deficient retinas (Supplementary Figure S6E-G). We comment on these results on page 8.

This manuscript is focused on how c-Src controls developmental angiogenesis in the retina. This is novel, as c-Src was thought to be required specifically for vascular permeability. We are currently working on additional manuscripts to investigate how the loss of c-Src, related SFK Yes and Fyn, and upstream mediators VEGFR2-Y949/TSAd, alter the blood-retinal barrier and blood-brain barrier. We have included some preliminary experiments to reveal that c-Src-deficient retinas may show reduced leakage of a 10kDa tracer at the vascular front of the retina but these results are too preliminary to include in the manuscript.

© 2020. Published by The Company of Biologists under the terms of the Creative Commons Attribution License (https://creativecommons.org/licenses/by/4.0/).

Tight junctions are known to play a role in the control of CNS vasculature integrity. It is important to note that this manuscript has only investigated adherens junctions and not tight junctions. We chose to focus on adherens junctions due to the known role of c-Src in regulating these VE-cadherin-comprised cell-cell adhesions, and the established role of VE-cadherin turnover in sprouting angiogenesis. We cannot discount the idea that tight junctions may be compromised, altering barrier integrity.

To perform a complete analysis by injecting both wildtype and c-Src deficient mice with different dextrans would be very time consuming, as experiments are technically challenging.

Mice at a range of ages (P3-P23) are required to be anaesthetised, injected with dextran via cardiac perfusion and then survive 15 minutes to allow for efficient dextran perfusion. While we agree that investigating the effects on vascular permeability upon loss of c-Src is important, we feel that these are beyond the scope of this manuscript as we would need to spend quite some time on becoming technically proficient and getting stable results. Indeed, these experiments can comprise an entire project in itself, with such a manuscript recently published in Neuron (Chow, B. W., & Gu, C. Gradual Suppression of Transcytosis Governs Functional Blood-Retinal Barrier Formation. *Neuron*, 93(6), 1325–1333.e3 (2017). However, we have considered how vascular permeability may be affected in the discussion (page 12).

R1.3. *While a major focus is placed on proteins involved in cell-cell junctions (i.e., paracellular transport), it is important to also assess whether the transcellular transport is affected or not by loss of c-Src function in endothelial cells.*

Response: Investigation of c-Src in regulation of transcellular transport is of great interest, but as discussed in point R1.2, our manuscript is focused on the role of c-Src in developmental angiogenesis. We agree the transcytosis process is understudied and merits in-depth analyses in genetic models, in particular as the potential role of c-Src in transcytosis has not been investigated in endothelial cells.

One way to examine transcytosis would be to study Caveolin1 (Cav1), which is known to be required for endothelial cell caveolae formation (Drab, M. *et al.* Loss of caveolae, vascular dysfunction, and pulmonary defects in caveolin-1 gene-disrupted mice. *Science* 293, 2449– 2452 (2001). Indeed, Cav1 is a known target of c-Src, and loss of phospho-Cav1 impairs caveolae function (Zimnicka, A. M. *et al.* Src-dependent phosphorylation of caveolin-1 Tyr-14 promotes swelling and release of caveolae. *Molecular Biology of the Cell*, 27(13), 2090–2106 (2016). Therefore, hypothetically we could measure whether phospho-Cav1 is altered upon loss of c-Src in the retina. However, while Cav1 is required for formation of caveolae, the exact role of Cav1 (and caveolae), in transcytosis is controversial. Indeed, albumin transport across the endothelium requires Cav1 and caveolae, yet surprisingly, animals lacking Cav1 display *increased* albumin leakage in the retinal vasculature (Gu, X. *et al.* Loss of Caveolin-1 Causes Blood-Retinal Barrier Breakdown, Venous Enlargement, and Mural Cell Alteration. *The American Journal of Pathology*, 184(2), 541-555 (2014). Additionally, recent studies have shown no role for Cav1 and caveolae in transport across the endothelium in tumours (Sindhwani, S. *et al.* The entry of nanoparticles into solid tumours. *Nature Materials*, 1–17 (2020).

To comprehensively investigate whether transcellular transport is altered in c-Src-deficient retinas, we would need to perform extensive EM studies to examine vesicular trafficking at different time points and within different areas of the retina. Although we are interested in a possible role for c-Src-dependent Cav1 phosphorylation as we now discuss on page 12, this will be the focus of an independent study.

R1.4. *Since the data suggest that endothelial loss of c-Src causes increased vascular regression, it is important to confirm this observation by assessing whether regression of the hyaloid vessels is affected or not upon loss of c-Src gene function.*

Response: We have assessed the hyaloid plexus vasculature at P5 in tdTomato reporter mice (Madisen L *et al.* A robust and high-throughput Cre reporting and characterization system for the whole mouse brain. *Nat Neurosci* 13(1):133-40 (2010) to confirm efficient Cre activity in this vascular bed. Despite efficient tdTomato expression, we did not observe any apparent changes in the vasculature of this tissue. We have included these results in Supplementary Figure 3M-P, mentioned on page 6, method

© 2020. Published by The Company of Biologists under the terms of the Creative Commons Attribution License (https://creativecommons.org/licenses/by/4.0/).

on page 17.

While the hyaloid plexus is an interesting tissue to study, it is known to be a separate vascular bed to the retina, controlled by different processes (reviewed in Wang, Z. *et al.* Assessment and Characterization of Hyaloid Vessels in Mice. *Journal of Visualized Experiments*, (147) (2019). Indeed, the hyaloid plexus begins to regress as the retinal vasculature starts to mature (Lutty, G. A., McLeod, D. S. Development of the hyaloid, choroidal and retinal vasculatures in the fetal human eye. *Progress in Retinal and Eye Research*. 62, 58-76 (2018). In mice, the hyaloid plexus starts as a complex network of vessels at P0, which regresses through development until the tissue is devoid of vasculature at P21 (Ito, M., Yoshioka, M. Regression of the hyaloid vessels and pupillary membrane of the mouse. *Anatomy and Embryology*. 200 (4), 403-411 (1999). In contrast, the retina is devoid of vessels at P0, and the vasculature grows out via angiogenic sprouting from P0-P7, before forming a secondary vascular layer later during development (Stahl, A. *et al.* The mouse retina as an angiogenesis model. *Investigative Ophthalmology & Visual Science*. 51 (6), 2813-2826 (2010).

The mechanisms guiding vascular regression in the hyaloid plexus are controlled by Wnt- mediated programmed cell death (Lobov, I. B. *et al.* WNT7b mediates macrophage-induced programmed cell death in patterning of the vasculature. *Nature*. 437 (7057), 417-421 (2005). In contrast, apoptosis has not been implicated in regression of retinal vasculature (Watson, E. C. *et al.* Apoptosis regulates endothelial cell number and capillary vessel diameter but not vessel regression during retinal angiogenesis. *Development*, 143(16), 2973–2982. (2016)) and rather, regression and remodelling are largely controlled by endothelial cell migration (Franco, C. A. *et al.* Dynamic Endothelial Cell Rearrangements Drive Developmental Vessel Regression. *PLoS Biology*, 13(4) (2015). In agreement, we have not observed any changes in cell death in the retinas of c-Src-deficient mice, (Figure 3) and believe the phenotype in the mice is due to altered cell migration and adhesion upon loss of c-Src. Thus, perhaps it is not surprising that we see no changes in the hyaloid vasculature.

***R1.5**. Since previous studies have shown that mice with global deletion of c-Src develop osteopetrosis due to impaired osteoclast function, it is important to assess the indirect effects of c-Src deletion on macrophages. The latter play an important role in vessel regression/remodeling during development.*

Response: We have performed immunostaining to visualise macrophages using an F4/80 antibody, co-stained with Collagen IV, to determine any association with these cells and empty matrix sleeves. We did not observe any changes in the number of macrophages in wildtype versus c-Src-deficient mice, or any changes in where macrophages localised in the retina with respect to empty CollIV sleeves. These results are shown in Figure 3O-Q and discussed on page 7-8. We agree this was an excellent point from reviewer 1, given the recent publication from He and colleagues (He *et al.*, The Cdh5-CreERT2 transgene causes conditional Shb gene deletion in hematopoietic cells with consequences for immune cell responses to tumors. *Sci Rep*. May 17;9(1):7548. (2019).

***R1.6**. Does loss of c-Src function affect pericyte recruitment to developing blood vessels as well?*

Response: We have performed immunostaining with pericyte marker NG2 and quantified whether there was reduced pericyte association with the Isolectin B4-positive vasculature. We did not observe any changes in pericyte coverage. These results are shown in Figure 3G-I and discussed on page 7.

***R1.7**. Angiogenic sprouting process and mechanisms in the retina is different form that in tracheal and metatarsal explants as the cellular components involved are different. Therefore, caution must be taken when extrapolating data obtained in these two different biological systems.*

Response: We agree with this statement, and have added a comment to emphasise this on page 6.

***R1.8**. The role of integrins is discussed in the discussion section of the paper but no data has been provided concerning the role of integrins in cell-matrix interactions in c-Src-deficient endothelial cells especially since cell-matrix interactions are affected by loss of c-Src function.*

Response: We have now included data to try and address which integrin may be involved in c-Src mediated cell-matrix adhesion, by growing cells on different substrates (shown in Figure 7). According to the literature, fibronectin should preferentially engage integrin $\alpha5\beta1$ and vitronectin

© 2020. Published by The Company of Biologists under the terms of the Creative Commons Attribution License (https://creativecommons.org/licenses/by/4.0/).

should engage $\alpha v\beta 3$ (references included in manuscript on pages 10 and 11). Figure 7 reveals that growing c-Src-deficient cells on vitronectin (Figure 7G, H) decreases both focal adhesion number (Figure 7I) and focal adhesion density (Figure 7J), whereas growing cells on fibronectin does not result in changes to focal adhesions upon loss of c-Src (Figure 7C-F). Interestingly, no change was observed in unstimulated cells in p-Paxillin (Supplementary Figure 8). These results are discussed in the results section (page 10-11), and discussion (page 14).

The results also support our hypothesis that $\alpha v\beta 3$ integrin is likely to be involved upstream of c-Src, and not $\beta 1$ integrin, given we do not detect changes in VE-cadherin morphology. However, to conclusively identify which integrin is engaged upstream of c-Src, experiments with specific blocking antibodies are required, as fibronectin can also engage $\alpha v\beta 3$ (Plow, E. F. *et al.* Integrin function in vascular biology. *Current Opinion in Hematology*, 21(3), 241–247 (2014) and vitronectin is known to engage other integrins such as $\alpha v\beta 1$, $\alpha v\beta 5$ and $\alpha 11b\beta 3$ (Felding-Habermann, B., & Cheresh, D. A. Vitronectin and its receptors. *Current Opinion in Cell Biology*, 5(5), 864–868 (1993). Therefore, we are careful state our results are indicative, not conclusive, of the integrins involved in this pathway.

R1.9. *Since c-Src is an intracellular protein and since its global deletion increases vascular permeability according to the literature, it is unclear why its endothelial-specific deletion had no effects on paracellular permeability of blood vessels.*

Response: We agree and have ensured that no statements making this claim are present in the manuscript. In addition, discussion on vascular permeability controlled through paracellular and transcellular mechanisms are included in the discussion, as mentioned in point R1.2 and R1.3 (page 12).

Reviewer 2 Advance Summary and Potential Significance to Field:

The manuscript by Schimmel and colleagues addresses the regulation of angiogenesis in the postnatal retina by the kinase c-Src. The authors propose that c-Src controls vessel growth through cell-matrix interactions and not through changes in vascular integrity.
The main findings of this study are: (1) c-Src is required for sprouting angiogenesis in retina by regulating focal adhesion assembly and cell-matrix adhesion, (2) c-Src has no overt effect on endothelial cell-cell adhesion, and (3) c-Src signaling acts differently on adherens junctions and focal adhesions.
These results improve our understanding of the mechanisms controlling vascular growth and, given the important roles of Src singling in many different processes, findings should be of interest to many researchers.

We thank the reviewer for their positive comments.

R2.1. *Overall, data quality and presentation are good, but insight into the underlying mechanism is limited. It is also noted that the manuscript could be improved by editing (e.g. see first sentence of the abstract) and the Discussion should be shortened by taking out aspects that are of peripheral relevance.*

Response: We thank the reviewer for these constructive comments. We have re-structured the abstract. We have also removed 2 paragraphs from the discussion of peripheral relevance, on the role of c-Src in compartmentalised endothelial locations (were previously on page 13 and page 14), which was also required to fit the manuscript to within the word limit with the new information from revisions.

Specific criticism:
R2.1. *Conditional deletion of c-Src in endothelial cells results in deletion of exons 7-9 and a frame shift mutation in exon 10. It would be important that authors check whether a truncated protein is formed after c-Src gene deletion, which may act as a dominant negative, kinase- deficient form. The antibody used in this study to detect c-Src was raised against epitopes 82- 169 of chicken v-Src and should therefore recognize a potential truncated variant comprised of SH3 and SH2 domains.*

© 2020. Published by The Company of Biologists under the terms of the Creative Commons Attribution License (https://creativecommons.org/licenses/by/4.0/).

Response: This is a very important point and we thank the reviewer for their suggestion. If a truncated form of c-Src is produced in the *c-Src$^{fl/fl}$;Cdh5CreERT2* mice, we should observe a band using our antibody at approximately 15.6kDa by western blot. Using endothelial cells isolated from lungs, where we observed a significant decrease in full length c-Src (and therefore efficient tamoxifen mediated Cre-activity, Fig 1), we do not observe a band at 15.6kDa. Therefore, we can conclude that we do not have a dominant negative, truncated form of c-Src. This data is shown in Supplementary Figure 2C and discussed on page 5.

R2.2. *While the role of c-Src in sprouting angiogenesis is well addressed, phospho-Src immunostaining is not shown in any experiment in vitro or in vivo. This information is crucial for understanding the role of Src in angiogenesis. How is active Src distributed between tip and stalk cells? Is it polarized and concentrated at the leading edge of tip cells? Is there is localized heterogeneity (as shown in vitro by Vera RH et al, Tissue Engineering, 2009) or differential expression in vessel subtypes?*

Response: Endothelial cells within the same tissue and across vascular beds are known to display extraordinary heterogeneity, therefore this is a very interesting line of study. We have now included results of phospho-c-Src immunostaining in cultured HUVEC in a sprouting scratch assay (Supplementary Figure 4). c-Src is not expressed at high levels in cells at the leading edge, but rather, seems to be slightly enriched in trailing cells (Supplementary Figure 4A-E). These data are in line with those from Figure 1 and 2, which demonstrate c-Src expression is not required for tip cell formation or identity. However, when we focused on localised expression of c-Src within endothelial cells at the sprouting front, we can see enrichment of p-c-Src at points within the cell which appear as FAs (Supplementary Figure 4C). Unfortunately, due to lack of c-Src-specific antibodies, we cannot co-stain with focal adhesion markers, but we are following up this line of study using tagged, lentiviral constructs, which will be the focus of future studies. Interestingly, within the monolayer, c-Src is enriched at cell-cell junctions (Supplementary Figure 4D), in line with our previous work and that of others, which revealed c-Src can regulate adherens junction activity in certain settings. These results are discussed on page 7 and 10.

While we agree *in vivo* retinal staining with a phospho-c-Src antibody would be ideal to further study heterogeneity in a more biologically relevant system, there are no antibodies that work for immunostaining which recognise active mouse c-Src that do not cross react with other Src family kinases, such as Yes and Fyn, which are both expressed strongly in endothelial cells. To truly examine c-Src activity, not only localisation of phospho-c-Src, we are employing endothelial Src-biosensor mice (Wang, Y. *et al*. Visualizing the mechanical activation of Src. *Nature, 434*(7036), 1040–1045 (2005). However, these experiments are the focus of follow- up studies.

R2.3. *The authors should provide detailed descriptions on how quantitation was done. Without this information, one cannot properly assess whether EC proliferation was unaffected in the c-Src mutant retinal plexus. As the role of Src in the regulation of cell proliferation was already shown, the authors should provide a deeper analysis of proliferation in different vascular beds, such as the capillary vascular plexus and the vein region. As mentioned above, it would be helpful to correlate these results with the localization of phosphorylated c-Src.*

Response: Images in Figure 3A-B are representative of where proliferation analysis was performed – at the vascular front region of the retina in the capillary plexus. Each set of quantifications (Collagen IV analysis, VE-cadherin phosphorylation analysis and phospho- Paxillin analysis) were performed at the same area: the vascular front in the plexus region, taking care to avoid areas around the artery or the vein. Unfortunately, due to lack of antibodies, we cannot directly correlate this to p-c-Src activity, as mentioned in R2.2.

Studies have shown that at P6, endothelial cell proliferation is largely confined to the vascular front and the venous region, being most pronounced at the distal perivenous capillaries (i.e. capillary plexus) (Ehling, M. *et al*. Notch controls retinal blood vessel maturation and quiescence. *Development*,140(14), 3051–3061 (2013). AND Xu, C. *et al*. Arteries are formed by vein-derived endothelial tip cells. *Nature Communications*, 1–11 (2019). Indeed, the area we have chosen for analysis of proliferation is standard in the angiogenesis field, as shown in Figure 5 of Pitulescu *et al*. (Pitulescu, M. E. *et al*. Inducible gene targeting in the neonatal vasculature and analysis of retinal angiogenesis in mice. *Nature Protocols*, *5*(9), 1518–1534 (2010). Therefore, we strongly believe this

© 2020. Published by The Company of Biologists under the terms of the Creative Commons Attribution License (https://creativecommons.org/licenses/by/4.0/).

is the most relevant area of the retina in which to analyse cell proliferation upon loss of c-Src. A statement clarifying where image analysis was performed has been added to the materials and methods (page 18).

We have also added a paragraph to the materials and methods describing how focal adhesion image analysis was performed (page 18).

R2.4. The authors found that c-Src has no effect on tip cell identity and does not regulate sprouting. Furthermore, deletion of c-Src resulted in reduced vessel stability in the angiogenic front area but also in the vascular plexus below (as indicated in Fig. 3J, K). The authors should provide the information about the regions where quantitations were done. In light of the vascular phenotype, it is not overly surprising that VE-cadherin in junctions of the angiogenic front area was not altered. Instead, the authors might want to carefully analyse VE-cadherin positive adherens junctions and VE-cadherin phosphorylation in the plexus region with increased collagen IV + empty sleeves.

Response: As stated in response to R2.3, the VE-cadherin phosphorylation analysis was performed at the vascular front, which is also where the empty Collagen IV sleeve analysis was performed. We have included a new Supplementary Figure 7, showing very high magnification of the sprouting front stained with Collagen IV, to reveal clear empty sleeves, commented within page 8. All images quantified were imaged in an identical fashion to the representative images shown, with the vascular front of the retina parallel to the image edge.

R2.5. Murine lung ECs were immunoselected using CD31 beads and these cells are likely to represent a mixture of different EC populations. To rule out completely that c-Src plays no role in VE-cadherin phosphorylation (which might have been overlooked by diluting the effect on c-Src using mixed EC populations) and internalization, the authors could investigate VE- cadherin phosphorylation in a wound assay using primary cells such as HUVECs. The pVEC 658 blot could be added to Fig. 4H.

Response: We agree that ECs isolated from lungs are indeed a mixed population, therefore western blot analysis will not reveal changes in heterogeneity upon loss of c-Src. This is why we have shown data on *in vivo*, phospho-VE-cadherin staining (Figure 4J-S) and performed VE-cadherin pattern analysis, as a proxy for VE-cadherin phosphorylation, as published by Bentley et al., NCB, 2014 (Figure 4A-G). This analysis was performed at the vascular front of the retina. We did not observe changes in VE-cadherin patterning in these heterogeneous, *in vivo* cell populations.

We did not originally include pVEC658 in Figure 4 as this antibody from the Dejana lab does not work for western blot. However, we have now included this data, using a commercially available antibody for pVEC 658 (from Invitrogen, added to materials and methods on page 15). Again, there was no significant difference in pVEC658 in c-Src-deficient lysates derived from lung endothelial cells (Figure 4H, I), reinforcing our hypothesis that VE-cadherin phosphorylation is not altered by loss of c-Src. These results are mentioned on page 8.

We have tried to perform immunostaining in a HUVEC scratch assay using all of our phospho- VEC antibodies. Unfortunately, none of the antibodies (from the Dejana lab or from Invitrogen) work for immunocytochemistry on HUVECs (being validated only for western blot or for use in mouse tissue), thus we were unable to effectively perform these experiments.

R2.6. In Figure 6 A and B pc-SrcY416/ c-Src total were increased in si-Src knockdown cells, maybe as compensatory effect, as suggested by the authors. This antibody is not entirely specific for pSrc 416 and detects other activated tyrosine kinases (as is also stated by the manufacturer). Therefore, the authors should validate antibody specificity by confirming that levels of pSrc are reduced when cells are treated with a Src family inhibitor, such as PP2.

Response: The reviewer has touched on one of the challenges of working with Src family kinases - most of the reagents display some degree of cross-reactivity with other members of the family. We have tested the majority of SFK antibodies which are commercially available, and have found reagents used within this study to be largely specific for c-Src, as opposed to Yes and Fyn. We have included data here for the reviewer where we have treated HUVEC with a c-Src inhibitor, Saracatinib, which we have previously validated to reduce c-Src activity (Gordon *et al.*, Science

© 2020. Published by The Company of Biologists under the terms of the Creative Commons Attribution License (https://creativecommons.org/licenses/by/4.0/).

Signalling, (2016). When stimulated with FCS, to ensure robust phosphorylation of c-Src, we see a decrease in p-c-Src in cells treated with Saracatinib compared to DMSO control, reduced to levels observed in unstimulated cells. We also stained HUVEC that were transfected with labelled c-Src lentivirus and observed increased staining intensity in cells with over-expression (OE) compared to neighbours. These results demonstrate that the antibody we have used to detect p-c-Src does indeed recognise when levels of c-Src are reduced or induced. We have also confirmed that our total c-Src antibody used throughout the manuscript is highly specific, with no cross-reactivity for other SFK, YES or FYN.

***R2.7**. The fact that c-Src regulates paxillin phosphorylation in vivo and the formation of focal adhesions is interesting. The main message of this study is that c-Src regulates sprouting by regulating focal adhesion assembly and cell-matrix adhesion. The authors nicely discuss potential mechanisms, which require further investigation. It was previously shown that not only focal adhesion formation but also fibronectin fibrillogenesis and actin organization depend on the phosphorylation state of paxillin (Zeidel-Bar R. et al., JCS, 2007). Culture of cells on glass without coating by matrix molecules might be therefore problematic for same of the assays.*

Response: We agree that culturing of cells can lead to problematic results. Therefore, we believe our conclusive results showing reduced phospho-Paxillin *in vivo* in the retina (Figure 7) are the most robust, conclusive evidence c-Src is involved in FA assembly.

All of our experiments investigating c-Src and FAs were done on matrix coated slides (fibronectin or vitronectin), which we have now emphasised in the figure legends for figures 6 and 7 and on pages 10-11 and 14.

The idea that c-Src itself could be regulating deposition of matrix by endothelial cells is interesting, and we thank the reviewer for bringing this to our attention. We have investigated whether fibronectin deposition is altered upon loss of c-Src when cells are grown on glass, as shown in Supplementary Figure 9. Despite reduction of c-Src staining intensity, we did not observe any changes in fibronectin deposition as analysed by immunostaining. This suggests, that in these settings, c-Src is not regulating matrix deposition by endothelial cells. These results are discussed on page 11.

***R2.8**. In Fig. 6, it is surprising and, based on the statements in the introduction, a bit counter-intuitive that VEGF stimulation does not lead to the activation of Src or FAK relative to the 0min time point. Please explain.*

Response: The endothelial cells in this assay were grown on fibronectin coated slides. Therefore, it is possible we did not see a change in c-Src or FAK activity due to matrix substrate conditions. As commented on page 9, we also did not see changes in VEGFR2 phosphorylation or internalisation upon VEGF stimulation when c-Src was decreased or increased, suggesting VEGF signaling occurs independently of c-Src in this setting. This would support the hypothesis that c-Src and FAK phosphorylation are not significantly increased upon VEGF stimulation under these conditions. We have added this comment to page 10.

Second decision letter

MS ID#: DEVELOP/2019/185405

MS TITLE: c-Src controls stability of sprouting blood vessels in the developing retina independently of cell-cell adhesion through focal adhesion assembly.

AUTHORS: Lilian Schimmel, Daisuke Fukuhara, Mark Richards, Yi Jin, Patricia Essebier, Emmanuelle Frampton, Marie Hedlund, Elisabetta Dejana, Lena Claesson-Welsh, and Emma Gordon
ARTICLE TYPE: Research Report

© 2020. Published by The Company of Biologists under the terms of the Creative Commons Attribution License (https://creativecommons.org/licenses/by/4.0/).

I am happy to tell you that your manuscript has been accepted for publication in Development, pending our standard ethics checks.

Reviewer 1

Advance summary and potential significance to field

Advances made in this paper are in the findings of the importance of c-Src signaling in cell-matrix interactions rather than cell-cell interactions during sprouting angiogenesis. c-Src is clearly important in the process of endothelial cell guidance and tip cell anastomosis and these effects do not seem to be specific to the retinal vascular bed since ex vivo studies with metatarsal explants showed the same effects observed in the retina. More importantly, the study rules out an effect of endothelial c-Src on endothelium integrity and vascular leakage. More in depth mechanistic analyses will be needed to investigate the molecular effectors downstream of c-Src that regulate the stabilization of new sprouts.

Comments for the author

I have no additional comments.

Reviewer 2

Advance summary and potential significance to field

This manuscript shows that c-Src regulates endothelial sprouting and cell-matrix interactions. It sheds new light on the function of c-Src and contains some surprising findings. in the stages of postnatal development investigated, c-Src is not essential for cell junction integrity. The phenotype of EC-specific Src mutants is relatively mild even though there is no compensatory up regulation of other Src family kinases.
Given the many known roles of c-Src in the regulation of cell behaviour, this manuscript improves our understanding of Src function in endothelial cells.

Comments for the author

The authors have addressed all my questions and the revised manuscript is much improved. I have no further questions or concerns.

© 2020. Published by The Company of Biologists under the terms of the Creative Commons Attribution License (https://creativecommons.org/licenses/by/4.0/).
